# Gut Microbiota Markers in Obese Adolescent and Adult Patients: Age-Dependent Differential Patterns

**DOI:** 10.3389/fmicb.2018.01210

**Published:** 2018-06-05

**Authors:** Federica Del Chierico, Francesca Abbatini, Alessandra Russo, Andrea Quagliariello, Sofia Reddel, Danila Capoccia, Romina Caccamo, Stefano Ginanni Corradini, Valerio Nobili, Francesco De Peppo, Bruno Dallapiccola, Frida Leonetti, Gianfranco Silecchia, Lorenza Putignani

**Affiliations:** ^1^Human Microbiome Unit, Bambino Gesù Children’s Hospital, IRCCS, Rome, Italy; ^2^Department of Medical Surgical Sciences and Biotechnologies, Faculty of Pharmacy and Medicine, Bariatric Center of Excellence IFSO-EU, Sapienza University, Rome, Italy; ^3^Department of Experimental Medicine, Section of Endocrinology, Sapienza University, Rome, Italy; ^4^Pediatric General Surgery Unit, Bambino Gesù Children’s Hospital, IRCCS, Rome, Italy; ^5^Gastroenterology Unit, Department of Clinical Medicine, Sapienza University, Rome, Italy; ^6^Department of Pediatrics, Sapienza University, Rome, Italy; ^7^Hepatogastroenterology and Nutrition Unit, Bambino Gesù Hospital, Rome, Italy; ^8^Scientific Directorate, Bambino Gesù Children’s Hospital, IRCCS, Rome, Italy; ^9^Parasitology Unit, Bambino Gesù Children’s Hospital, IRCCS, Rome, Italy

**Keywords:** gut microbiota, obesity, bacterial markers, metabolic pathways, dysbiosis

## Abstract

Obesity levels, especially in children, have dramatically increased over the last few decades. Recently, several studies highlighted the involvement of gut microbiota in the pathophysiology of obesity. We investigated the composition of gut microbiota in obese adolescents and adults compared to age-matched normal weight (NW) volunteers in order to assemble age- and obesity-related microbiota profiles. The composition of gut microbiota was analyzed by 16S rRNA-based metagenomics. Ecological representations of microbial communities were computed, and univariate, multivariate, and correlation analyses performed on bacterial profiles. The prediction of metagenome functional content from 16S rRNA gene surveys was carried out. Ecological analyses revealed a dissimilarity among the subgroups, and resultant microbiota profiles differed between obese adolescents and adults. Using statistical analyses, we assigned, as microbial markers, *Faecalibacterium prausnitzii* and *Actinomyces* to the microbiota of obese adolescents, and *Parabacteroides*, Rikenellaceae, *Bacteroides caccae*, Barnesiellaceae, and *Oscillospira* to the microbiota of NW adolescents. The predicted metabolic profiles resulted different in adolescent groups. Particularly, biosynthesis of primary bile acid and steroid acids, metabolism of fructose, mannose, galactose, butanoate, and pentose phosphate and glycolysis/gluconeogenesis were for the majority associated to obese, while biosynthesis and metabolism of glycan, biosynthesis of secondary bile acid, metabolism of steroid hormone and lipoic acid were associated to NW adolescents. Our study revealed unique features of gut microbiota in terms of ecological patterns, microbial composition and metabolism in obese patients. The assignment of novel obesity bacterial markers may open avenues for the development of patient-tailored treatments dependent on age-related microbiota profiles.

## Introduction

There has been a dramatic increase in the prevalence of obesity over the last few decades, with the total number of overweight and obese individuals increasing globally from 857 million in 1980 to 2.1 billion in 2013 (Nicolucci and Reimer, 2016). Of particular concern is the 47.1% increase in the prevalence of childhood obesity between 1980 and 2013 (Nicolucci and Reimer, 2016).

In the United States, there has been a dramatic increase in obesity and extreme obesity from the period 1988–1994 to the period 2013–2014: in particular, obese adolescents increased from 10.5 to 20.6%, and extremely obese adolescents from 2.6 to 9.1% (Ogden et al., 2016).

According to a report by the Organization for Economic Co-operation and Development, in 2014 in Italy, approximately 1 out of 10 adults was obese, while 40% were overweight (including obesity). With regard to children, 36% of boys and 34% of girls were overweight or obese (Organization for Economic Co-operation and Development, 2014).

Childhood obesity tends to persist into adulthood. Therefore, obesity in children implies both immediate and imminent co-morbidity risks, many of which are similar to those observed in adults (e.g., asthma and cognitive impairment in childhood, diabetes, heart disease, several cancers, respiratory diseases, mental health, and reproductive disorders later in life) (Adair et al., 2016). Obesity in childhood is a public health challenge that will require low cost, non-invasive and targeted interventions (Keating et al., 2011; Kalra et al., 2012; Ogden et al., 2014). To prevent long-term consequences of obesity on health, bariatric surgery has also been advocated in adolescents (Leslie et al., 2009). However, less than 1% of all weight loss procedures are currently performed in adolescents, though this percentage may increase with time (Burguera et al., 2007).

Studies on obese humans and animals have provided some of the most persuasive indications of the intricate and mutually beneficial interactions between bacteria and humans (Cani and Delzenne, 2009; Ridaura et al., 2013). Gut bacteria is involved in the regulation of host energy metabolism and body mass (Clarke et al., 2012). Specifically, the intestinal microbiota was shown to be a key factor in the degradation of non-digestible nutriments, such as plant polysaccharides and fibers (Chiu et al., 2014). Moreover, the gut microbiota influences host physiology and contributes to nutrition and metabolic health (Koh et al., 2016). Indeed, modulation of the gut microbiota by consumption of fiber, antibiotic treatment or fecal transplantation can have positive effects on inflammation and insulin sensitivity (Robertson et al., 2005; Cani et al., 2008). An altered gut microbiota has been observed in obesity and T2D (Larsen et al., 2010; Karlsson et al., 2013; Le Chatelier et al., 2013), and in particular decreased species and gene richness have been linked to adiposity, dyslipidemia, and insulin resistance (Cotillard et al., 2013).

Genome sequencing experiment of several gut organisms evidenced the presence of enzymatic capacities intrinsic to gut bacteria lacking in the human genome. This is the evidence that microorganisms make it possible to digest diet components that would be otherwise inaccessible to the host (Xu et al., 2003).

A primary end point of this prospective study was to investigate the composition of the gut microbiota in obese adolescents versus adults, and in normal weight (NW) health teenagers and adults. A secondary end point was to identify specific microorganisms and bacterial metabolic pathways that correlated with obesity, focusing on possible age-related biomarkers.

## Materials and Methods

### Patient and Control Recruitment

Obese patients were prospectively enrolled at the obesity clinic. Twenty adult patients with an indication for bariatric surgery were screened following the guidelines of the Italian Society for Obesity Surgery (Sicob^1^). Twenty-five adolescent patients were enrolled for bariatric surgery according to the guidelines of ESPGHAN (Nobili et al., 2015).

To obtain an age-matched control-case study, 12 NW healthy adolescents (CTRL_ado) and 12 NW healthy adults (CTRL_adult) were enrolled as volunteers during the same period at the OPBG Metagenomics Human Microbiome Unit and at the Clinical Medicine Department of Umberto I Hospital, respectively. Anamnestic and clinical data for obese patients and CTRLs are reported in **Table 1**. From each subjects/patients a single stool sample was collected and stored at -80°C until the end of collection.

**Table 1:** Anamnestic and clinical data of patients and CTRLs.

	ob_ado	ob_adult	CTRL_ado	CTRL_adult
N. of subjects	25	20	12	12
Gender (female/male)	14/11	14/6	8/4	8/4
Age^a^ (mean ± *SD*)	16.16 ± 2.08	40.65 ± 11.65	16.92 ± 1.08	33.75 ± 10.59
BMI^b^ (mean ± *SD*)	44.82 ± 6.09	41.02 ± 7.10	20.69 ± 0.88	21.02 ± 1.51
Intragastric balloon positioning	9	–	–	–
Impaired glucose metabolism^c^	6 IGT	1 DMT2^d^/4 IGT	–	–
Blood hypertension^e^	–	10 yes	–	–

*^*a*^Age in years; ^*b*^BMI in kg/m^*2*^; ^*c*^impaired glucose metabolism: no, yes, impaired glucose tolerance (IGT); ^*d*^DMT2: diabetes mellitus type 2; ^*e*^blood hypertension: no, yes.*

Co-morbidity assessment, and psychological and nutritional counseling were performed in an outpatient setting. Inclusion criteria were: Caucasian race; age 13–19 years for obese adolescents (ob_ado) and 20–65 years for obese adults (ob_adult); body mass index (BMI) between 30 and 60 kg/m^2^; eligibility for bariatric surgery (Sleeve Gastrectomy). Exclusion criteria were: histological positivity to *Helicobacter pylori*; intake of corticosteroids, antibiotics, or pre-probiotics in the previous 2 months; intake of vitamin E or fish oil in the previous 2 months; chronic gastrointestinal diseases or syndromes (e.g., IBD and IBS); previous bariatric surgery (intragastric balloon excluded).

The criteria for healthy CTRL enrolment were: BMI between 18.5 and 24.9 kg/m^2^ for adults (World Health Organization [WHO], 2000) and BMI between the 5th and the 85th percentile of BMI-for-age, for adolescents (Ogden et al., 2002), absence of chronic diseases; absence of gastrointestinal infections in the previous 2 months, no antibiotic and pre-probiotic intake in the previous 2 months, omnivorous diet.

This study was carried out in accordance with the recommendations of the OPBG Ethics Committee (Protocol No. 768.12) for adolescent patients and by Umberto I hospital Ethics Committee (Protocol No. 1003/13) for adult patients. All subjects gave written informed consent in accordance with the Declaration of Helsinki.

Arterial Hypertension was defined according to the modified NCEP ATP-III criteria. Blood pressure (mmHg) was registered after 5 min of rest using an electronic auscultatory blood pressure recorder with an appropriately sized cuff, and with the patient sitting in the upright position. Three measurements were taken, and the average of the second and third measurements was recorded and used in the analysis. Patients were considered as affected by hypertension when values were over 135/80 mmHg.

Impaired glucose metabolism was defined according to the American Diabetes Association (ADA) criteria (American Diabetes Association, 2009). Patients underwent an oral glucose tolerance test (OGTT) with glycemia measurement at baseline and 30, 60, 90, and 120 min after the ingestion of 75 g of glucose. Patients were defined as Normal Glucose Tolerant (NGT) with glycemia values under 140 mg/dl at 120 min of OGTT; impaired glucose tolerant (IGT) with glycemia values between 140 and 200 mg/dl at 120 min of OGTT; type 2 diabetes patients (DMT2) with glycemia values over 200 mg/dl.

### Genomic DNA Extraction, Pyrosequencing, and Quantitative Analysis of the Microbiome Composition

Genomic DNA was isolated from the entire set of 69 stool samples, using the QIAamp DNA Stool Mini Kit (Qiagen, Germany). DNA quantity and quality was assessed by NanoDrop^TM^ 2000/2000c spectrophotometer (Thermo Scientific, Wilmington, MA, United States). For the 16S rRNA-based metagenomics, the V1–V3 regions (520 bp) of the 16S rRNA gene were amplified to obtain bacterial library using universal bacterial primers (FW 5′-GAGTTTGATCNTGGCTCA G-3′, RV 5′-GTNTTACNGCGGCKGCTG-3′). Primers were barcoded by eight unique nucleotide sequences (Roche 454 Life Sciences, Branford, CT, United States). To guarantee high specificity, sensitivity and accuracy of PCR reaction, a Hi-Fi PCR Taq polymerase (FastStart^TM^ High Fidelity PCR System, dNTPack, Roche Diagnostics, Mannheim, Germany) was employed. To limit the per base PCR error rates and chimeric sequences 40 cycles of PCR, 5 min of extension time, a low template concentrations (1 ng) conditions were applied (Lahr and Katz, 2009). Each samples was submitted to pyrosequencing reaction on a 454-Junior Genome Sequencer (Roche 454 Life Sciences, Branford, CT, United States), according to manufacturer’s instructions.

The 454 Amplicon signal processing was used as first result filtering, hence by QIIME 1.8.0 software the sequences were analyzed (Caporaso et al., 2010). After demultiplexing, reads with an average quality score lower than 25, shorter than 300 bp and with an ambiguous base calling were excluded from the analysis. Sequences that passed the quality filter were denoised (Reeder and Knight, 2010) and singletons were excluded. The denoised sequences were chimera-checked by *identify_chimeric_seqs.py* using either Blast_fragments and ChimeraSlayer^2^ approaches. The operational taxonomic units (OTUs) defined by a 97% of similarity were picked and the representative sequences were submitted to PyNAST for the sequence alignment (Caporaso et al., 2010), and to UCLUST for sequence clustering (Edgar, 2010). The database for OTUs matching was greengenes (v 13.8). After rarefying, the α- and β-diversity and the ANOSIM tests were carried out by QIIME software, using *alpha_rarefaction.py, beta_diversity_through_plots.py, compare_categories.py* scripts; the *group_significance.py* script was used to perform OTUs Kruskal–Wallis test (Navas-Molina et al., 2013).

### Statistical Analysis

Operational taxonomic units present in less than 25% of the samples were removed prior to applying statistical analysis. At the end of the filtering process, the taxa taken into consideration for subsequent statistical analyses were reduced to 47/222 OTUs.

Statistical tests [Shapiro–Wilk test, ANOVA, least significant difference (LSD) test, Mann–Whitney *U*-test, receiver operating characteristic (ROC), discriminant analysis (DA), principal component analysis (PCA), Wilks’ Lambda test, Spearman’s correlations] were performed by IBM SPSS statistic software version 21.

To predict metagenome functional content from 16S rRNA gene surveys, Picrust v 1.1.0 tool have been applied (Langille et al., 2013) and to obtain the KEGG (Kyoto Encyclopedia of Genes and Genomes) pathways^3^ we analyzed the function prediction by HUMAnN2 v0.99 program (Abubucker et al., 2012). Furthermore, to find KEGGs biomarkers associated with adolescent obese and CTRL conditions, a linear discriminant effect size (LeFse) analysis have been performed (α value = 0.05, logarithmic LDA score threshold = 2.0) (Segata et al., 2011).

### MG Data Open Access Repository

Sequencing reads and the associated metadata are available at NCBI: Bioprojects: PRJNA356507, gut metagenomic profile from obese patients; PRJNA280490, gut metagenomic profile from healthy subjects^4^.

## Results

### Microbiota Profiles in Obese Patients and in Healthy Controls

A total of 139,783.00 sequencing reads were obtained from 69 fecal samples, with a mean value of 2,025.84 sequences per sample. We identified an overall of 222 OTUs, grouped in 14 phylum and 79 families.

To assess the overall differences of microbial community structures in patients and CTRLs, we measured ecological parameters based on alpha-diversity (ChaoI, Shannon indexes).

The highest mean value of the ChaoI index was obtained for the CTRL_ado group (242.28), followed by CTRL_adult (180.23), ob_ado (166.03), and ob_adult (149.28) groups (Supplementary Figure S1A). The mean Shannon index values are similar amongst the four groups (Supplementary Figure S1B).

**FIGURE 1:**
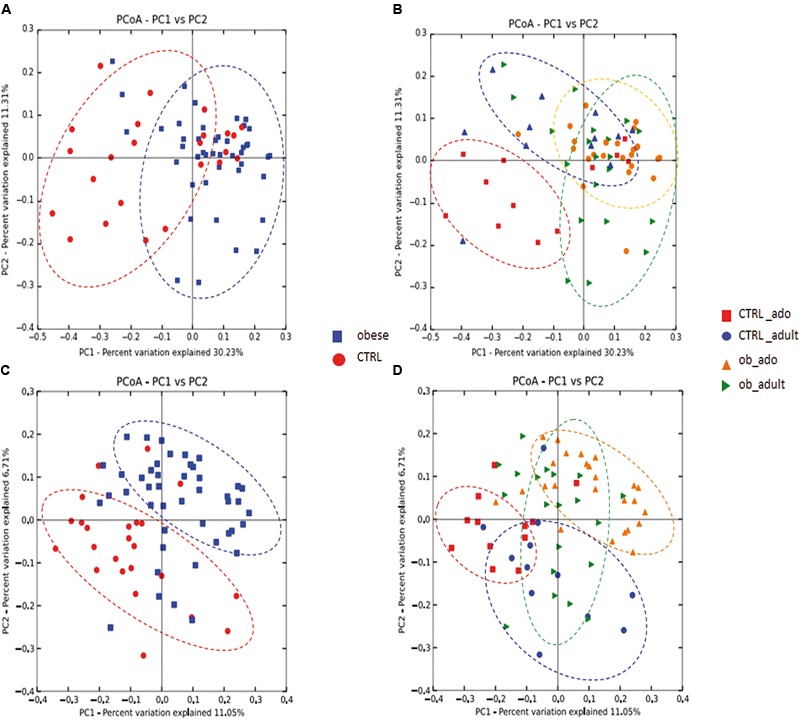
Principal coordinates analysis (PCoA) plot of obese and CTRL groups **(A–C)**, and of CTRL_ado, CTRL_adult, ob_ado, and ob_adult groups **(B–D)**. The plots show the first two principal coordinates (axes) for PCoA using a weighted **(A,B)** and unweighted **(C,D)** UniFrac algorithm.

To determine the differences between microbial community structures in obese patients and CTRLs, we calculated β-diversity. Our results showed that, by weighted UniFrac analysis, the first coordinate (PC1) explained the 30.23% of the intersample variance (*p* = 0.01; **Figure 1A**), while the unweighted UniFrac analysis explained the 11.05% in obese versus CTRL group (*p* = 0.01; **Figure 1C**). Stratifying samples by age, we found that the two adolescent groups (ob_ado and CTRL_ado) formed more defined and separated clusters than the adult groups (*p* = 0.01; **Figures 1B,D**).

**FIGURE 2:**
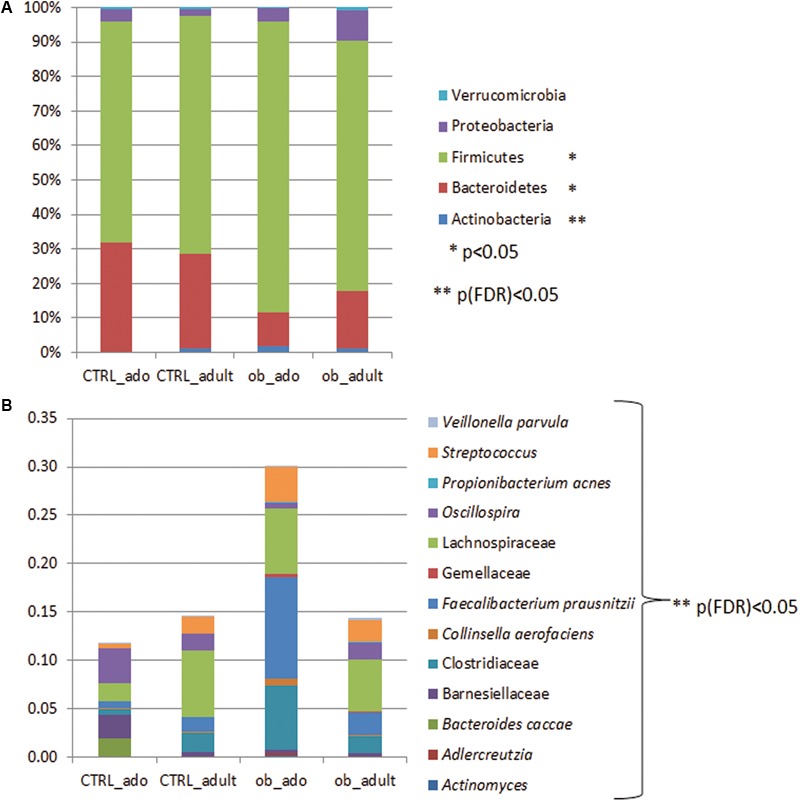
Bar chart representing Kruskal–Wallis test results on operational taxonomic units (OTUs) grouped in phyla **(A)** and in families/species **(B)** of the CTRL_ado, CTRL_adult, ob_ado, ob_adult groups. Each column in the plot represents a group, and each color in the column represents: **(A)** the percentage of relative abundance for each OTU; **(B)** the values of relative abundance for each OTU.

Grouping OTUs at phylum level, we took into consideration the relative abundances of the five major phyla (e.g., Actinobacteria, Bacteroidetes, Firmicutes, Proteobacteria, and Verrucomicrobia). Applying the Kruskal–Wallis test on the relative abundances of phyla for the four groups, we observed statistically significant differences in the distributions of Actinobacteria, Bacteroidetes, and Firmicutes (*p* < 0.05). However, a false discovery rate (FDR) correction of *p*-values defined Actinobacteria as the only significant taxon differentially describing the four groups (**Figure 2A** and Supplementary Table S1).

Particularly, the distribution of Actinobacteria and Bacteroidetes were statistically significant for the ob_ado/ob_adult couple, with Actinobacteria levels being higher in the ob_ado group, and Bacteroidetes levels being uppermost in the ob_adult group. Actinobacteria, Bacteroidetes, and Firmicutes were statistically significant for adolescent obese versus CTRL (Supplementary Table S2).

At OTU level the Kruskal–Wallis test revealed the following statistically significant comparisons: *Actinomyces, Adlercreutzia, Bacteroides caccae*, Barnesiellaceae, Clostridiaceae, *Collinsella aerofaciens, Faecalibacterium prausnitzii*, Gemellaceae, Lachnospiraceae, *Oscillospira, Propionibacterium acnes, Streptococcus*, and *Veillonella parvula* ([p]FDR < 0.05; **Figure 2B** and Supplementary Table S3).

By the Mann–Whitney *U*-test, applied to the above mentioned 13 OTUs, we identified that *Actinomyces*. *Adlercreutzia* and *C. aerofaciens* and *F. prausnitzii* were higher in ob_ado respect to ob_adult (*p* < 0.05; **Table 2**). Interestingly, a high number of comparisons were statistically significant in the pairwise ob_ado *versus* CTRL_ado, particularly *Actinomyces, Adlercreutzia*, Clostridiaceae, *C. aerofaciens, F. prausnitzii*, Lachnospiraceae, *P. acnes, Streptococcus*, and *V. parvula* were higher in the ob_ado group, while *B. caccae*, Barnesiellaceae, and *Oscillospira* were higher in CTRL_ado group (*p* < 0.05) (Supplementary Table S4). Finally, *F. prausnitzii* and *P. acnes* were higher in ob_adult respect to CTRL_adult.

**Table 2:** Mann–Whitney *U*-test on OTUs at family/species level for the CTRL_ado, CTRL_adult, ob_ado, ob_adult groups.

	Mean of relative abundances											
OTUs	ob_ado	ob_adult	ob_ado	CTRL_ado	ob_ado	CTRL_adult	ob_adult	CTRL_ado	ob_adult	CTRL_adult	CTRL_ado	CTRL_adult
*Actinomyces*^a,b,c^	0.002	0.000	0.002	0.001	0.002	0.000	0.000	0.001	0.000	0.000	0.001	0.000
*Adlercreutzia*^a,b^	0.003	0.000	0.003	0.000	0.003	0.001	0.000	0.000	0.000	0.001	0.000	0.001
*Bacteroides caccae*^b,d,f^	0.001	0.000	0.001	0.020	0.001	0.000	0.000	0.020	0.000	0.000	0.020	0.000
Barnesiellaceae^b,d,f^	0.004	0.004	0.004	0.027	0.004	0.006	0.004	0.027	0.004	0.006	0.027	0.006
Clostridiaceae^b,c,d^	0.079	0.021	0.079	0.007	0.079	0.021	0.021	0.007	0.021	0.021	0.007	0.021
*Collinsella aerofaciens*^a,b,c^	0.009	0.001	0.009	0.001	0.009	0.001	0.001	0.001	0.001	0.001	0.001	0.001
*Faecalibacterium prausnitzii*^a,b,c,d,e^	0.111	0.027	0.111	0.007	0.111	0.017	0.027	0.007	0.027	0.017	0.007	0.017
Gemellaceae	0.003	0.000	0.003	0.000	0.003	0.000	0.000	0.000	0.000	0.000	0.000	0.000
Lachnospiraceae^b,d,f^	0.079	0.061	0.079	0.020	0.079	0.079	0.061	0.020	0.061	0.079	0.020	0.079
*Oscillospira*^b,c,d,f^	0.008	0.021	0.008	0.043	0.008	0.019	0.021	0.043	0.021	0.019	0.043	0.019
*Propionibacterium acnes*^b,c,d,e^	0.001	0.001	0.001	0.000	0.001	0.000	0.001	0.000	0.001	0.000	0.000	0.000
*Streptococcus*^b,d,f^	0.040	0.031	0.040	0.004	0.040	0.020	0.031	0.004	0.031	0.020	0.004	0.020
*Veillonella parvula*^b^	0.002	0.005	0.002	0.000	0.002	0.000	0.005	0.000	0.005	0.000	0.000	0.000

*For each group the mean of relative abundances is reported in columns. ^*a*^Significative *p*-value for ob_ado versus ob_adult; ^*b*^significative *p*-value for ob_ado versus CTRL_ado; ^*c*^significative *p*-value for ob_ado versus CTRL_adult; ^*d*^significative *p*-value for ob_adult versus CTRL_adult; ^*e*^significative *p*-value for ob_adult versus CTRL_adult; ^*f*^significative *p*-value for CTRL_ado versus CTRL_adult.*

### Detecting Microbial Biomarkers in Obese Patients and Healthy Controls

To define a model based on the capability of OTUs to discriminate the four groups of study participants, we performed a discriminant analysis (DA) based on univariate ANOVAs, Fisher’s coefficient and leave-one-out classification.

**FIGURE 3:**
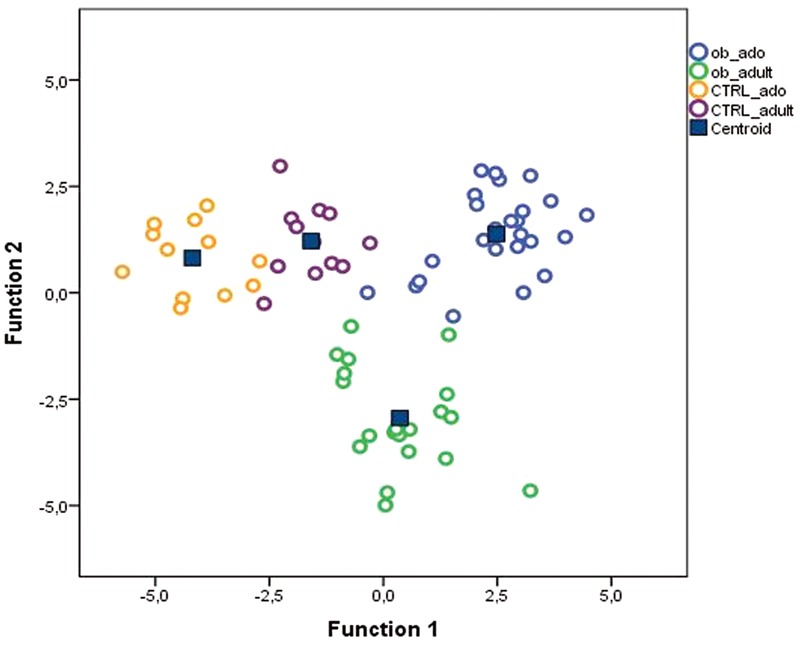
Canonical discriminant plot. Scatter plot of canonical discriminant analysis (DA) based on univariate ANOVA and Fisher’s coefficient applied to all OTUs of samples belonging to CTRL_ado, CTRL_adult, ob_ado, and ob_adult groups.

A DA showed that 98.6% of the original grouped cases were correctly classified, and the canonical discriminant plot revealed a clear separation between obese and CTRL groups (**Figure 3** and Supplementary Table S5). In particular, the Figure Shows a clear separation between the samples belonging to the four groups, with most of the samples being close to the centroid of the group of belonging, although a lower separation was observed between obese groups (ob_ado and ob_adult) (**Figure 3**). However, applying a cross-validation (CV) test, we found that only 34.8% of cases were correctly classified, revealing a low capability of the entire OTUs set to discriminate the four groups (Supplementary Table S5). For this reason, using a *p* < 0.05 filter (from a Wilks’ Lambda test) we selected only 12 OTUs (Supplementary Figure S2 and Supplementary Table S6) and tested their discriminatory power in correctly classifying groups by applying the average area under the ROC.

The area under the ROC curve (AUROC) for the ob_ado group was 0.75 for *F. prausnitzii*, and 0.735 for *Actinomyces*; for the CTRL_ado group, it was 0.706 for *Parabacteroides*, 0.731 for Rikenellaceae, 0.781 for *B. caccae*, 0.848 for Barnesiellaceae, and 0.725 for *Oscillospira* (Supplementary Table S7). For ob_adult and CTRL_adult groups, AUROC values were <0.7 and then considered not accurate in discriminating study groups (Swets, 1988).

**FIGURE 4:**
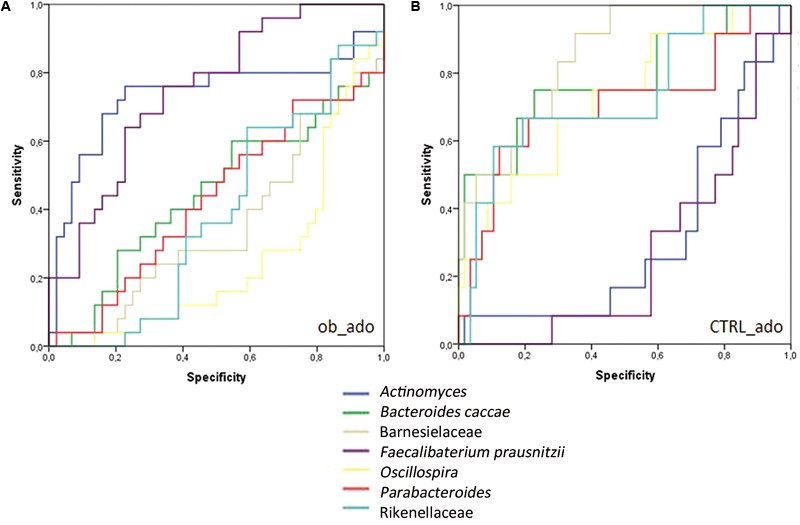
Receiver operating characteristic (ROC) curve plots. The areas under the ROC curves (AUROC) represent the specificity and sensitivity of the seven selected OTUs (AUROC > 0.7) able to discriminate ob_ado **(A)** and CTRL_ado **(B)** groups.

These results indicated that *F. prausnitzii* and *Actinomyces* were accurate in discriminating the ob_ado group, and *Parabacteroides*, Rikenellaceae, *B. caccae*, Barnesiellaceae, and *Oscillospira* allowed us to discriminate the CTRL_ado group (**Figure 4**). However, none of the OTUs was able to discriminate adult groups. These results were confirmed and validate by DA and CV analyses (Supplementary Table S8).

### Correlations Between Clinical/Anthropometric Characteristics and Bacterial Abundance

To evaluate correlations amongst bacteria, and clinical and anthropometric characteristics (e.g., age, gender, BMI, the presence of diabetes and hypertension), we selected Spearman’s rho cut-off values, also taking into account *r* > 0.4, *r* < -0.4 (*p* < 0.05).

For ob_ado patients, Spearman’s correlation analysis revealed that only Erysipelotrichaceae negatively correlated with the BMI. *Anaerostipes* negatively correlated with age, while *P. distasonis* positively correlated with age. Conversely, diabetes was positively correlated with the presence of *Bacteroides, Parabacteroides, P. copri* and *P. distasonis*, while no OTU correlated with hypertension and gender features (Supplementary Figure S3 and Supplementary Table S9).

For the ob_adult group, *Coprococcus* positively correlated with the BMI, while *Bacteroides fragilis, Dehalobacterium, Lachnospira*, and Enterobacteriaceae negatively correlated with the BMI. *Blautia* and Lachnospiraceae negatively correlated with gender. Moreover, diabetes was positively correlated with the presence of Bacteroidaceae, *P. distasonis*, Lachnospiraceae and *Blautia*, but was negatively correlated with *Prevotella, Lachnospira* and Enterobacteriaceae. Hypertension correlated with *Sutterella, Butyricimonas*, and *Paraprevotella*. No OTU correlated with age (Supplementary Figure S3 and Supplementary Table S9). Correlation analysis in the CTRL_ado group revealed a positive correlation between age and the BMI, and a negative correlation between age and *F. prausnitzii*. Surprisingly, gender positively correlated with the presence of *P. acnes*, Erysipelotrichaceae and Enterobacteriaceae. Moreover, *F. prausnitzii* was negatively correlated with the BMI (Supplementary Figure S3 and Supplementary Table S10).

In the CTRL_adult group, no correlation between OTUs and age was found, while *A. muciniphila* negatively correlated with BMI. Furthermore, gender negatively correlated with *Blautia*, and positively with *Clostridium* and *Sutterella* (Supplementary Figure S3 and Supplementary Table S10).

### Metabolic Pathway Predictions

A total of 20 KEGG pathways were generated using the composition of the gut microbiota based on PICRUSt in adolescent versus adult obese, of which lipopolysaccharide, folate, vitamin B6, inositol phosphate, flavonoid biosynthesis pathways were higher in adolescent, while arginine and proline, amino and nucleotide sugar, starch and sucrose galactose metabolism pathways were higher in adults (Supplementary Figure S4).

Moreover, 60 differential metabolic patterns differentially expressed resulted in the ob_ado versus CTRL_ado comparison. Thirty-four KEGG pathways were significantly upregulated in the ob_ado group while 26 were significantly upregulated in CTRL_ado group. Among them, primary bile acid biosynthesis, steroid acids biosynthesis, fructose and mannose metabolism, glycolysis/gluconeogenesis, galactose metabolism, butanoate metabolism, and pentose phosphate metabolism were for the majority associated to obese, while glycan biosynthesis and metabolism, secondary bile acid biosynthesis, steroid hormone biosynthesis, and lipoic acid metabolism were associated to NW adolescents (Supplementary Figure S5). In the comparison between adults we obtained 10 pathways associated obese and 9 associated to NW condition. Among pathways associated to adult obese were taurine and hypotaurine, beta-alanine, propanoate, tryptophan metabolism, while nitrogen, amino and nucleotide sugars, alanine, aspartate, and glutamate metabolism were associated to NW (Supplementary Figure S6).

## Discussion

In this study, we characterized gut microbiota profiles in obese adolescents and adults, and compared these to NW healthy age-matched study participants.

The gut microbiota may be considered a microbial metabolic organ because of its influence on the regulation of energy uptake from the diet, its involvement in host metabolism, and the release of gut hormones (Hullar and Lampe, 2012). Several studies on the gut microbiome have shown obese individuals harbor less diverse bacterial communities than lean individuals (Turnbaugh et al., 2009; Pihl et al., 2016). In particular, Le Chatelier et al. (2013) showed that individuals with low bacterial richness presented with a marked overall adiposity, insulin resistance and dyslipidemia compared with individuals showing high bacterial richness.

Studies based on both animal and human models have described an altered OTU composition in fecal microbiota related to obesity (Turnbaugh et al., 2006; Zhang et al., 2009; Clarke et al., 2012; Chiu et al., 2014). Moreover, these studies reported conflicting results on the microbiota’s composition, leading to confusing conclusions on microorganisms’ roles, both in terms of pathogenicity and protective bacteria, the latter being helpful for the identification of tailored probiotics. The large inter-individual variation in microbiota composition related to obesity is probably due to the employment of different research methodologies and the involvement of participants with different backgrounds (food habits and ethnicity) (Aguirre and Venema, 2015).

It has been found that gut microbiota in humans and animal models of obesity is reduced in abundance of Bacteroidetes with a proportional increase in Firmicutes phylum, however, these results have been contradicted by other studies reporting divergent ratios between these two phyla (Duncan et al., 2008; Abdallah Ismail et al., 2011; Harris et al., 2012).

Our study focused not only on differences in the composition of gut microbiota of obese patients compared to CTRLs, but also on changes between young and adult study participants in order to verify an age-related association between microbes and obesity.

The microbiota profile in obese adolescents resulted differentially composed respect to obese adults, leading to suppose a synergic role of age and obesity in microbiota composition. However, the main differences in microbiota composition were evidenced comparing adolescent obese and NW. In fact, the OTU-based model assigned the microbial markers *F. prausnitzii* and *Actinomyces* to the ob_ado group, and *Parabacteroides*, Rikenellaceae, *B. caccae*, Barnesiellaceae, and *Oscillospira* to the CTRL_ado group.

*Faecalibacterium prausnitzii* is one of the most common species in the gastrointestinal tract of adults consuming a Western diet (Scott et al., 2013) and its presence has been associated with lower success in weight loss diets (Le Chatelier et al., 2013). Furthermore, *F. prausnitzii* has a key role in host metabolism as it allows the fermentation of unabsorbed carbohydrate. The presence of *F. prausnitzii* in the gut of obese adolescents may lead to increased energy recovery from unabsorbed carbohydrate that would not otherwise contribute to dietary energy intake (Balamurugan et al., 2010).

In our study, *Actinomyces* was also found to be related to the gut microbiota of obese adolescents. *Actinomyces* species are part of the normal, resident microbiota of the mouth. They contributed to different plaque related diseases (Moore and Moore, 1994), and correlated with a glucose diet (Matee et al., 1993). Moreover, in two case reports, *Actinomyces* were found responsible for gastric actinomycosis as a complication after gastric bypass for morbid obesity (Fernández-Aceñero et al., 2004; Baierlein et al., 2007).

Amongst the OTUs that correlated with the gut microbiota of NW healthy participants, we found *Parabacteroides*, and *B. caccae*, both belonging to the Bacteroidetes phylum. *P. distasonis* is prominently found in the gut of healthy individuals (Xu et al., 2007); and studies in humans and in mouse models demonstrated a positive correlation between Bacteroidetes and weight loss (Cani et al., 2008; Nadal et al., 2009).

Regarding the association of Barnesiellaceae with the NW group, Chiu et al. (2014) reported higher levels of *Barnesiella* (belonging to Barnesiellaceae family) in control individuals compared to obese patients.

Finally, in our previous study on non-alcoholic fatty liver patients, we reported a higher abundance of *Oscillospira*, Rikenellaceae, *Parabacteroides, B. fragilis*, in CTRLs compared to patients, consistently with present data (Del Chierico et al., 2016).

Changes in functional capacity of gut microbiota indicated by KEGG pathways were different between adolescent and adult obese. In particular, adolescent obese microbiota was enriched by lipopolysaccharide metabolism, that have been recognized to initiate the inflammation-related processes associated with the onset of obesity and insulin resistance (Boulangé et al., 2016). Vitamin B6 is required for the synthesis of fat from carbohydrate and protein (Zhou and Zhou, 2014). However, inositol phosphate, folate, flavonoid biosynthesis pathways seems to have a role against the obesity. In particular, inositol plays a role in glucose metabolism control, reducing BMI (Saleem and Rizvi, 2017); folate treatment improved insulin resistance and endothelial dysfunction in patients with metabolic syndrome (Pinhas-Hamiel et al., 2006); flavonoids seem to have potential benefit role against obesity (Kawser Hossain et al., 2016).

In adults, obese microbiome compared with NW resulted enriched in pathways involved in the initial steps in breaking down indigestible dietary polysaccharides, including starch/sucrose metabolism, galactose metabolism (Turnbaugh et al., 2006), but also glutamate and carbohydrate metabolism.

As already evidenced in the microbiota profile description, the main differences in KEEG pathway profiles were obtained comparing adolescents obese and NW. The NW microbiota of adolescents is significantly enriched in glycan biosynthesis and metabolism pathways. Mostly of glycans arise from rich fiber diet and host mucosal secretions. Microbes ferments indigestible glycans producing short chain fatty acids (SCFAs), which are nutrients for colonocytes and enterocytes (Koropatkin et al., 2012).

Greater primary bile acid biosynthesis was associated to obese adolescents, while secondary bile acid biosynthesis was associated to NW subjects. Primary bile acids (i.e., cholate and chenodeoxycholate), are deconjugated by several gram-positive bacterial species, such as Lactobacilli (Begley et al., 2006). After deconjugation, additional microbial modifications give rise to the formation of secondary bile acids (Jones et al., 2008), which is only carried out by a minor population of gram-positive anaerobic *Clostridium* species (Kitahara et al., 2004). Interestingly, bile acids, particularly secondary ones, influence energy expenditure and glucose homeostasis via their effects on gluconeogenesis, insulin secretion and insulin sensitivity (Vrieze et al., 2014).

Furthermore, steroid hormone biosynthesis and lipoic acid metabolism pathways were associated to NW subjects. Regulation of some key proteins in adipose tissues by sex steroid hormones may also be a mechanism for the treatment and/or prevention of obesity (Mayes and Watson, 2004). In addition, chronic administration of lipoic acid improves glucose tolerance and skeletal muscle glucose transport in the obese rat (Saengsirisuwan et al., 2004).

Notably, the obese adolescent microbiome is significantly enriched for KEGG pathways involved in the fructose and mannose metabolism, glycolysis/gluconeogenesis, galactose metabolism, butanoate metabolism, and pentose phosphate metabolism. Also in a diet-induced obesity study, the high-fat/high-sugar Western diet was associated the phosphotransferase system, fructose and mannose metabolism, and glycolysis/gluconeogenesis pathways, due to a blooming of Firmicutes with reduction on Bacteroidetes (Turnbaugh et al., 2008). Furthermore, the study of Kim and Bae (2016) highlighted that the mucosal and luminal viromes of obese mice were significantly enriched with temperate phages associated with the Bacilli, Negativicutes and Bacteroidia classes. Particularly, the temperate phages from the Bacteroidia class encoded stress and niche-specific functions advantageous to bacterial host adaptation showing the role of phage in the obese microbiota shaping (Kim and Bae, 2016). Furthermore, in mice transplanted by microbiomes from obese twins, higher expression of microbial genes involved in the pentose phosphate pathway was highlighted (Ridaura et al., 2013). Additionally, microbiome of obese mice encoded many enzymes involved in breaking down indigestible dietary polysaccharides, including KEGG pathways for galactose metabolism and butanoate metabolism (Turnbaugh et al., 2006).

This finding suggested that the gut microbiota from obese subjects could influence metabolites that characterize the obese state.

Therefore, the gut microbiota appears to function as an ecological unit, the composition and local diversity of which are largely determined by niche-driven processes (Jeraldo et al., 2012). External factors, such as diet and lifestyle, but also diseases, such as obesity, diabetes or metabolic syndromes, alter the niche landscape by supplying novel nutrients, leading to fluctuations in the microbiota structure.

## Conclusion

**FIGURE 5:**
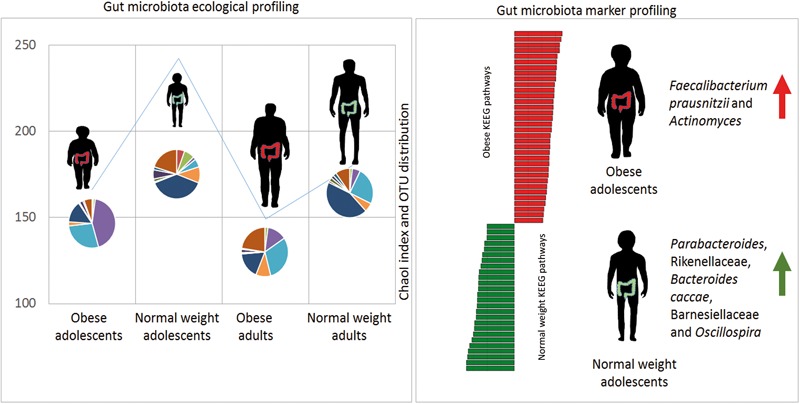
Gut microbiota model in obese and normal weight (NW) controls. Gut microbiota ecological richness (ChaoI index) results are lower in obese patients with respect to NW study participants. The microbiota profile (OTU distribution) is completely different in the four groups. By applying statistical analyses, it has been possible to generate a model able to associate microbial and metabolic markers with obese and NW adolescents.

This study revealed unique characteristics in ecological diversity, composition, and metabolic pathways of the gut microbiota of obese adolescents and adults compared to NW adolescents and adults. Nevertheless, future studies are need to valid these results in a more numerous cohort of patients. The proposed model associates specific microbial biomarkers to adolescent obese and to NW microbiota profiles (**Figure 5**). These microbial targets represent differential markers in microbiota profiling analyses. New clinical interventions, based on microbiota modulation, may be developed for the prevention and treatment of childhood-onset obesity. The markers identified in this study may therefore be considered in the process of developing new, specifically age-dependent probiotics targeted to obesity.

## Author Contributions

FDC: healthy subject enrollment and sample collection, data analysis and interpretation, and manuscript writing. FA: patient and healthy subject recruitment, sample collection, and manuscript writing. AR: data acquisition. AQ: data analysis and manuscript revising. SR: data analysis. DC: patient and healthy subject recruitment, sample collection, and manuscript writing. RC: patient recruitment and sample collection. SGC: study conception. VN: manuscript revising. FDP: patient recruitment and sample collection. BD: manuscript revising. FL: patient and healthy subject recruitment and sample collection. GS: study conception and design. LP: study conception and design and manuscript revising.

## Conflict of Interest Statement

The authors declare that the research was conducted in the absence of any commercial or financial relationships that could be construed as a potential conflict of interest. The reviewer PS and handling Editor declared their shared affiliation.

## Footnotes

1 www.sicob.org

2 http://qiime.org/scripts/identify_chimeric_seqs.html

3 http://huttenhower.sph.harvard.edu/humann2

4 http://www.ncbi.nlm.nih.gov/bioproject/?term=
