# Supplementary material for: Gut Microbiota Markers in Obese Adolescent and Adult Patients: Age-Dependent Differential Patterns
Source: Front Microbiol. 2018 Jun 5;9:1210. doi: 10.3389/fmicb.2018.01210 (PMC5996250; doi:10.3389/fmicb.2018.01210)
Supplement: Supplementary file 1 [file Data_Sheet_1.doc]

# Title

# Gut microbiota markers in obese adolescent and adult patients: age-dependent differential patterns

Federica Del Chierico^1^, Francesca Abbatini^2^, Alessandra Russo^1^, Andrea Quagliariello^1^, Sofia Reddel^1^, Danila Capoccia^3^, Romina Caccamo^4^, Stefano Ginanni Corradini^5^, Valerio, Nobili^6,7^, Francesco De Peppo^4^, Bruno Dallapiccola^8^, Frida Leonetti^3^, Gianfranco Silecchia^2^ and Lorenza Putignani^1,9*^

^1^Human Microbiome Unit, Bambino Gesù Children’s Hospital, IRCCS, Rome, Italy;

^2^Department of Medical Surgical Sciences and Biotechnologies, Faculty of Pharmacy and Medicine, Bariatric Center of Excellence IFSO-EU, Sapienza University, Rome, Italy;

^3^Department of Experimental Medicine, Section of Endocrinology, Sapienza University, Rome, Italy;

^4^Pediatric General Surgery Unit, Bambino Gesù Children’s Hospital, IRCCS, Rome, Italy;

^5^Gastroenterology Unit, Department of Clinical Medicine, Sapienza University, Rome, Italy;

^6^Pediatric Department - University "La Sapienza" Rome;

^7^Hepatogastroenterology and Nutrition Unit - Bambino Gesu' Hospital Rome;

^8^Scientific Directorate, Bambino Gesù Children’s Hospital, IRCCS, Rome, Italy;

^9^Parasitology Unit, Bambino Gesù Children’s Hospital, IRCCS, Rome, Italy.

**Correspondence:** Lorenza Putignani, [lorenza.putignani@opbg.net](mailto:lorenza.putignani@opbg.net)

**Table S1**. Kruskal-Wallis test on OTUs at phylum level for the CTRL_ado, CTRL_adult, ob_ado, ob_adult groups. In columns are reported mean, p and FDR_p values of each phylum for all the groups.

| **OTU** | **ob_ado** | **ob_adult** | **CTRL_ado** | **CTRL_adult** | **P** | **FDR_P** |
| --- | --- | --- | --- | --- | --- | --- |
| Actinobacteria | 0.0176 | 0.0111 | 0.0025 | 0.0096 | **0.0006** | **0.0052** |
| Bacteroidetes | 0.0963 | 0.1657 | 0.3138 | 0.2702 | **0.0423** | 0.1352 |
| Firmicutes | 0.8400 | 0.7234 | 0.6367 | 0.6792 | **0.0172** | 0.0690 |
| Proteobacteria | 0.0391 | 0.0879 | 0.0329 | 0.0195 | 0.1765 | 0.4034 |
| Verrucomicrobia | 0.0020 | 0.0075 | 0.0062 | 0.0044 | 0.5475 | 0.6653 |

**Table S2. U Mann-Whitney test on OTUs at phylum level for the CTRL_ado, CTRL_adult, ob_ado, ob_adult groups.**

|  |  | **Actinobacteria** | **Bacteroidetes** | **Firmicutes** | **Proteobacteria** | **Verrucomicrobia** |
| --- | --- | --- | --- | --- | --- | --- |
| ob_ado versus ob_adult | ob_ado | 0.0176 | 0.0963 | 0.8400 | 0.0391 | 0.0020 |
|  | ob_adult | 0.0111 | 0.1657 | 0.7234 | 0.0879 | 0.0075 |
|  | p value | **0.00** | 0.11 | **0.01** | 0.73 | 0.19 |
| ob_ado versus CTRL_ado | ob_ado | 0.0176 | 0.0963 | 0.8400 | 0.0391 | 0.0020 |
|  | CTRL_ado | 0.0025 | 0.3138 | 0.6367 | 0.0329 | 0.0062 |
|  | p value | **0.00** | **0.03** | **0.03** | 0.16 | 0.35 |
| ob_adult versus CTRL_adult | ob_adult | 0.0111 | 0.1657 | 0.7234 | 0.0879 | 0.0075 |
|  | CTRL_adult | 0.0096 | 0.2702 | 0.6792 | 0.0195 | 0.0044 |
|  | p value | 0.38 | 0.29 | 0.67 | 0.11 | 0.42 |

**Table S3**. Kruskal-Wallis test on OTUs at family/species level for the CTRL_ado, CTRL_adult, ob_ado, ob_adult groups. In columns are reported mean, p and FDR_p values of each statistically significant comparisons.

|  | **ob_ado** | **ob_adult** | **CTRL_ado** | **CTRL_adult** | **P values** | **FDR** |
| --- | --- | --- | --- | --- | --- | --- |
| *Actinomyces* | 0.0014 | 0.0002 | 0.0005 | 0.0000 | **0.0002** | **0.0099** |
| *Adlercreutzia* | 0.0024 | 0.0000 | 0.0000 | 0.0004 | **0.0020** | **0.0449** |
| *Bacteroides caccae* | 0.0007 | 0.0001 | 0.0185 | 0.0003 | **0.0007** | **0.0228** |
| *Barnesiellaceae* | 0.0034 | 0.0035 | 0.0248 | 0.0049 | **0.0009** | **0.0249** |
| *Clostridiaceae* | 0.0665 | 0.0178 | 0.0063 | 0.0191 | **0.0005** | **0.0189** |
| *Collinsella aerofaciens* | 0.0067 | 0.0012 | 0.0011 | 0.0012 | **0.0000** | **0.0010** |
| *Faecalibacterium prausnitzii* | 0.1051 | 0.0237 | 0.0068 | 0.0156 | **0.0011** | **0.0283** |
| Gemellaceae | 0.0027 | 0.0001 | 0.0002 | 0.0000 | **0.0035** | **0.0463** |
| Lachnospiraceae | 0.0676 | 0.0539 | 0.0183 | 0.0693 | **0.0001** | **0.0099** |
| *Oscillospira* | 0.0065 | 0.0182 | 0.0363 | 0.0166 | **0.0025** | **0.0463** |
| *Propionibacterium acnes* | 0.0013 | 0.0008 | 0.0002 | 0.0002 | **0.0029** | **0.0463** |
| *Streptococcus* | 0.0356 | 0.0220 | 0.0040 | 0.0174 | **0.0031** | **0.0463** |
| *Veillonella parvula* | 0.0012 | 0.0027 | 0.0000 | 0.0004 | **0.0025** | **0.0463** |

**Table S4. U Mann-Whitney test on OTUs at family/species level for the CTRL_ado, CTRL_adult, ob_ado, ob_adult groups.**

|  | ob_ado versus ob_adult | | | ob_ado versus CTRL_ado | | | ob_adult versus CTRL_adult | | |
| --- | --- | --- | --- | --- | --- | --- | --- | --- | --- |
|  | ob_ado | ob_adult | p value | ob_ado | CTRL_ado | p value | ob_adult | CTRL_adult | p value |
| *Actinomyces* | 0.0014 | 0.0002 | **0.03** | 0.0014 | 0.0005 | **0.01** | 0.0002 | 0.0000 | 0.14 |
| *Adlercreutzia* | 0.0024 | 0.0000 | **0.03** | 0.0024 | 0.0000 | **0.02** | 0.0000 | 0.0004 | 0.79 |
| *Bacteroides caccae* | 0.0007 | 0.0001 | 0.71 | 0.0007 | 0.0185 | **0.01** | 0.0001 | 0.0003 | 0.97 |
| Barnesiellaceae | 0.0034 | 0.0035 | 0.26 | 0.0034 | 0.0248 | **0.00** | 0.0035 | 0.0049 | 0.37 |
| Clostridiaceae | 0.0665 | 0.0178 | 0.07 | 0.0665 | 0.0063 | **0.00** | 0.0178 | 0.0191 | 0.09 |
| *Collinsella aerofaciens* | 0.0067 | 0.0012 | **0.00** | 0.0067 | 0.0011 | **0.04** | 0.0012 | 0.0012 | 0.97 |
| *Faecalibacterium prausnitzii* | 0.1051 | 0.0237 | **0.05** | 0.1051 | 0.0068 | **0.00** | 0.0237 | 0.0156 | **0.05** |
| Gemellaceae | 0.0027 | 0.0001 | 0.28 | 0.0027 | 0.0002 | 0.18 | 0.0001 | 0.0000 | 0.35 |
| Lachnospiraceae | 0.0676 | 0.0539 | 0.13 | 0.0676 | 0.0183 | **0.00** | 0.0539 | 0.0693 | 0.37 |
| *Oscillospira* | 0.0065 | 0.0182 | 0.11 | 0.0065 | 0.0363 | **0.00** | 0.0182 | 0.0166 | 0.82 |
| *Propionibacterium acnes* | 0.0013 | 0.0008 | 0.44 | 0.0013 | 0.0002 | **0.00** | 0.0008 | 0.0002 | **0.04** |
| *Streptococcus* | 0.0356 | 0.0220 | 0.48 | 0.0356 | 0.0040 | **0.00** | 0.0220 | 0.0174 | 0.85 |
| *Veillonella parvula* | 0.0012 | 0.0027 | 0.06 | 0.0012 | 0.0000 | **0.03** | 0.0027 | 0.0004 | 0.97 |

**Table S5. Discriminant analysis table based on 12 selected OTUs and 4 subject groups.**

|  | | **Groups** | **predicted group membership** | | | |
| --- | --- | --- | --- | --- | --- | --- |
|  |  |  | **ob_ado** | **ob_adult** | **CTRL_ado** | **CTRL_adult** |
| **Original** | % | **ob_ado** | 96 | 4 | 0 | 0 |
|  |  | **ob_adult** | 0 | 100 | 0 | 0 |
|  |  | **CTRL_ado** | 0 | 0 | 100 | 0 |
|  |  | **CTRL_adult** | 0 | 0 | 0 | 100 |
| **Cross-validated** | % | **ob_ado** | 36 | 32 | 20 | 12 |
|  |  | **ob_adult** | 15 | 45 | 20 | 20 |
|  |  | **CTRL_ado** | 33.3 | 8.3 | 16.7 | 41.7 |
|  |  | **CTRL_adult** | 8.3 | 33.3 | 25 | 33.3 |

**Table S6. Lambda di Wilks test on 47 selected OTUs at family/species level.**

|  | Lambda di Wilks | F | df1 | df2 | Sig. |
| --- | --- | --- | --- | --- | --- |
| Barnesiellaceae | 0.7206 | 8.4029 | 3 | 65 | **0.0001** |
| *Bacteroides* | 0.7713 | 6.4261 | 3 | 65 | **0.0007** |
| Lachnospiraceae | 0.7822 | 6.0347 | 3 | 65 | **0.0011** |
| *Oscillospira* | 0.8003 | 5.4076 | 3 | 65 | **0.0022** |
| *Parabacteroides distasonis* | 0.8528 | 3.7411 | 3 | 65 | **0.0152** |
| *Parabacteroides* | 0.8572 | 3.6108 | 3 | 65 | **0.0178** |
| *Actinomyces* | 0.8586 | 3.5678 | 3 | 65 | **0.0187** |
| *Dorea* | 0.8589 | 3.5603 | 3 | 65 | **0.0189** |
| *Butyricimonas* | 0.8671 | 3.3222 | 3 | 65 | **0.0251** |
| *Bacteroides caccae* | 0.8675 | 3.3100 | 3 | 65 | **0.0254** |
| *Faecalibacterium prausnitzii* | 0.8697 | 3.2475 | 3 | 65 | **0.0274** |
| Rikenellaceae | 0.8809 | 2.9284 | 3 | 65 | **0.0402** |
| *Adlercreutzia* | 0.8876 | 2.7440 | 3 | 65 | 0.0501 |
| *Anaerostipes* | 0.8927 | 2.6038 | 3 | 65 | 0.0593 |
| Mogibacteriaceae | 0.8942 | 2.5631 | 3 | 65 | 0.0623 |
| Clostridiaceae | 0.8949 | 2.5445 | 3 | 65 | 0.0637 |
| Christensenellaceae | 0.9004 | 2.3974 | 3 | 65 | 0.0761 |
| *Propionibacterium acnes* | 0.9022 | 2.3487 | 3 | 65 | 0.0806 |
| Ruminococcaceae | 0.9056 | 2.2588 | 3 | 65 | 0.0899 |
| *Collinsella aerofaciens* | 0.9155 | 1.9998 | 3 | 65 | 0.1227 |
| *Acidaminococcus* | 0.9204 | 1.8733 | 3 | 65 | 0.1428 |
| *Blautia* | 0.9276 | 1.6917 | 3 | 65 | 0.1775 |
| *Akkermansia muciniphila* | 0.9286 | 1.6671 | 3 | 65 | 0.1827 |
| *Dehalobacterium* | 0.9287 | 1.6641 | 3 | 65 | 0.1834 |
| *Phascolarctobacterium* | 0.9338 | 1.5365 | 3 | 65 | 0.2134 |
| Bacteroidaceae | 0.9365 | 1.4686 | 3 | 65 | 0.2313 |
| Erysipelotrichaceae | 0.9366 | 1.4678 | 3 | 65 | 0.2315 |
| *Prevotella* | 0.9418 | 1.3396 | 3 | 65 | 0.2692 |
| *Paraprevotella* | 0.9418 | 1.3384 | 3 | 65 | 0.2696 |
| *Granulicatella* | 0.9460 | 1.2364 | 3 | 65 | 0.3037 |
| *Streptococcus* | 0.9526 | 1.0789 | 3 | 65 | 0.3643 |
| *Turicibacter* | 0.9530 | 1.0684 | 3 | 65 | 0.3687 |
| *Prevotella copri* | 0.9533 | 1.0620 | 3 | 65 | 0.3714 |
| *Bacteroides fragilis* | 0.9546 | 1.0307 | 3 | 65 | 0.3849 |
| Enterobacteriaceae | 0.9561 | 0.9959 | 3 | 65 | 0.4004 |
| *Sutterella* | 0.9575 | 0.9627 | 3 | 65 | 0.4158 |
| Gemellaceae | 0.9594 | 0.9161 | 3 | 65 | 0.4382 |
| Coriobacteriaceae | 0.9599 | 0.9050 | 3 | 65 | 0.4436 |
| Enterococcus | 0.9697 | 0.6770 | 3 | 65 | 0.5693 |
| Coprococcus | 0.9702 | 0.6647 | 3 | 65 | 0.5768 |
| *Lachnospira* | 0.9714 | 0.6377 | 3 | 65 | 0.5935 |
| *Ruminococcus* | 0.9718 | 0.6288 | 3 | 65 | 0.5990 |
| *Clostridium* | 0.9724 | 0.6142 | 3 | 65 | 0.6083 |
| *Ruminococcus gnavus* | 0.9733 | 0.5938 | 3 | 65 | 0.6213 |
| *Veillonella parvula* | 0.9736 | 0.5878 | 3 | 65 | 0.6252 |
| *Eubacterium biforme* | 0.9772 | 0.5048 | 3 | 65 | 0.6803 |
| *Dialister* | 0.9910 | 0.1972 | 3 | 65 | 0.8980 |

**Table S7. Area under ROC curve (AUROC) of OTUs resulted statistically significant from Lambda Wilks test.**

| **Variables** | **ob_ado** | **ob_adu** | **CTRL_ado** | **CTRL_adu** |
| --- | --- | --- | --- | --- |
|  | **AUROC** | | | |
| *Actinomyces* | **0.735** | 0.491 | 0.310 | 0.326 |
| *Bacteroides caccae* | 0.450 | 0.410 | **0.781** | 0.428 |
| Barnesiellaceae | 0.351 | 0.492 | **0.848** | 0.404 |
| *Faecalibacterium prausnitzii* | **0.750** | 0.535 | 0.266 | 0.282 |
| Lachnospiraceae | 0.677 | 0.487 | 0.130 | 0.604 |
| *Oscillospira* | 0.250 | 0.572 | **0.725** | 0.573 |
| *Bacteroides* | 0.414 | 0.382 | 0.674 | 0.635 |
| *Butyricimonas* | 0.363 | 0.490 | 0.689 | 0.547 |
| *Dorea* | 0.521 | 0.405 | 0.468 | 0.635 |
| *Parabacteroides* | 0.432 | 0.464 | **0.706** | 0.455 |
| *Parabacteroides distasonis* | 0.463 | 0.397 | 0.655 | 0.553 |
| Rikenellaceae | 0.387 | 0.537 | **0.731** | 0.398 |

**Table S8. Discriminant analysis table based on 7 selected OTUs and 2 adolescent groups.**

| **groups** | | | **predicted group membership** | |
| --- | --- | --- | --- | --- |
|  |  |  | **ob_ado** | **CTRL_ado** |
| **Original** | % | ob_ado | 100.0 | 0.0 |
|  |  | CTRL_ado | 33.3 | 66.7 |
| **Cross-validated** | % | ob_ado | 100.0 | 0.0 |
|  |  | CTRL_ado | 50.0 | 50.0 |

**Table S9.** Spearman’s correlation table on OTUs and anamnestic and clinical data in ob_ado and in ob_adult groups.

|  |  |  |  |  |  | Actinobacteria | Actinobacteria | Actinobacteria | Actinobacteria | Actinobacteria | Bacteroidetes | Bacteroidetes | Bacteroidetes | Bacteroidetes | Bacteroidetes | Bacteroidetes | Bacteroidetes | Bacteroidetes | Bacteroidetes | Bacteroidetes | Bacteroidetes | Firmicutes | Firmicutes | Firmicutes | Firmicutes | Firmicutes | Firmicutes | Firmicutes | Firmicutes | Firmicutes | Firmicutes | Firmicutes | Firmicutes | Firmicutes | Firmicutes | Firmicutes | Firmicutes | Firmicutes | Firmicutes | Firmicutes | Firmicutes | Firmicutes | Firmicutes | Firmicutes | Firmicutes | Firmicutes | Firmicutes | Firmicutes | Firmicutes | Proteobacteria | Proteobacteria | Verrucomicrobia |
| --- | --- | --- | --- | --- | --- | --- | --- | --- | --- | --- | --- | --- | --- | --- | --- | --- | --- | --- | --- | --- | --- | --- | --- | --- | --- | --- | --- | --- | --- | --- | --- | --- | --- | --- | --- | --- | --- | --- | --- | --- | --- | --- | --- | --- | --- | --- | --- | --- | --- | --- | --- | --- |
|  | Age | Gender | BMI | Diabetes | Hypertension | *Actinomyces* | *Adlercreutzia* | *Collinsella aerofaciens* | Coriobacteriaceae | *Propionibacterium acnes* | Bacteroidaceae | *Bacteroides* | *Bacteroides caccae* | *Bacteroides fragilis* | Barnesiellaceae | *Butyricimonas* | *Parabacteroides* | *Parabacteroides distasonis* | *Paraprevotella* | *Prevotella* | *Prevotella copri* | *Acidaminococcus* | *Anaerostipes* | *Blautia* | Christensenellaceae | Clostridiaceae | *Clostridium* | *Coprococcus* | *Dehalobacterium* | *Dialister* | *Dorea* | *Enterococcus* | Erysipelotrichaceae | *Eubacterium biforme* | *Faecalibacterium prausnitzii* | Gemellaceae | *Granulicatella* | *Lachnospira* | Lachnospiraceae | Mogibacteriaceae | *Oscillospira* | *Phascolarctobacterium* | Rikenellaceae | Ruminococcaceae | *Ruminococcus* | *Ruminococcus gnavus* | *Streptococcus* | *Turicibacter* | *Veillonella parvula* | Enterobacteriaceae | *Sutterella* | *Akkermansia muciniphila* |
| **ob_ado** | **Spearman’s Rho** | | | | | | | | | | | | | | | | | | | | | | | | | | | | | | | | | | | | | | | | | | | | | | | | | | | |
| Age | 1 | 0.3 | 0.2 | 0.3 | 0.3 | 0 | -0.2 | -0.3 | 0 | -0.1 | 0 | 0 | 0.1 | -0.2 | -0.2 | 0 | 0.1 | **0.5** | 0.1 | 0 | 0.1 | -0.1 | **-0.5** | 0.2 | -0.1 | -0.1 | -0.2 | -0.2 | 0 | -0.4 | 0.2 | 0 | -0.2 | 0.1 | 0.1 | 0.1 | -0.1 | 0 | -0.2 | -0.1 | 0 | 0.1 | 0.2 | -0.1 | -0.1 | -0.2 | 0 | 0 | 0.3 | -0.2 | 0.2 | 0.3 |
| Gender | 0.3 | 1 | 0.3 | -0.1 | -0.2 | -0.1 | -0.3 | -0.2 | -0.2 | 0.2 | 0.2 | 0.1 | -0.1 | -0.2 | -0.1 | 0 | -0.3 | 0 | -0.1 | 0 | -0.1 | 0.2 | 0 | 0.2 | -0.1 | -0.1 | -0.4 | 0.1 | 0.1 | -0.2 | -0.2 | 0 | -0.3 | -0.3 | 0.1 | -0.1 | -0.2 | -0.1 | -0.1 | -0.4 | 0.1 | 0.1 | 0.3 | 0.1 | 0.3 | 0 | 0.2 | 0 | -0.1 | -0.3 | 0 | 0.2 |
| BMI | 0.2 | 0.3 | 1 | 0.2 | -0.2 | 0 | 0.2 | -0.1 | -0.2 | -0.1 | -0.1 | -0.2 | 0 | 0 | -0.1 | 0 | -0.1 | 0.2 | -0.1 | -0.2 | 0.3 | -0.2 | 0 | -0.2 | -0.1 | 0.2 | 0.2 | -0.4 | -0.2 | 0 | 0.1 | 0.3 | **-0.4** | 0.1 | -0.3 | 0.1 | 0.1 | -0.1 | -0.1 | -0.1 | -0.1 | -0.4 | 0.2 | 0 | 0 | 0.4 | 0.2 | 0.1 | 0 | 0 | -0.4 | 0.4 |
| Diabetes | 0.3 | -0.1 | 0.2 | 1 | 0.2 | 0.2 | 0.2 | 0.1 | 0.1 | -0.2 | 0.2 | **0.4** | 0.1 | 0 | 0.2 | 0.2 | **0.4** | **0.5** | 0.1 | 0.2 | **0.5** | 0.2 | 0 | -0.1 | 0.4 | -0.2 | -0.1 | -0.1 | 0.3 | 0.1 | 0.1 | 0.2 | 0 | 0.4 | 0 | 0.3 | 0.1 | -0.1 | 0 | 0.3 | 0.3 | 0 | 0 | 0 | 0.4 | -0.1 | 0.1 | -0.1 | 0.1 | 0.2 | 0 | 0.3 |
| Hypertension | 0.3 | -0.2 | -0.2 | 0.2 | 1 | 0.3 | 0.3 | 0.3 | 0.3 | -0.1 | -0.3 | -0.2 | -0.2 | -0.3 | -0.2 | -0.2 | 0.1 | 0.1 | -0.2 | 0.2 | -0.2 | -0.2 | -0.3 | 0.3 | -0.2 | -0.2 | -0.3 | 0.2 | -0.2 | 0 | 0.3 | -0.2 | 0.3 | -0.2 | 0.2 | 0.3 | 0.2 | 0.1 | -0.1 | 0.1 | 0.1 | 0.2 | -0.3 | 0 | -0.1 | -0.3 | 0.1 | -0.3 | 0.1 | 0.3 | 0.1 | -0.2 |
| **ob_ado** | **P values** | | | | | | | | | | | | | | | | | | | | | | | | | | | | | | | | | | | | | | | | | | | | | | | | | | | |
| Age |  | 0.1 | 0.3 | 0.2 | 0.1 | 0.9 | 0.3 | 0.2 | 0.9 | 0.7 | 0.8 | 0.9 | 0.8 | 0.3 | 0.5 | 0.9 | 0.6 | 0 | 0.6 | 1 | 0.7 | 0.5 | 0 | 0.4 | 0.7 | 0.7 | 0.4 | 0.3 | 0.8 | 0.1 | 0.3 | 0.9 | 0.3 | 0.5 | 0.6 | 0.8 | 0.8 | 0.9 | 0.2 | 0.7 | 0.8 | 0.7 | 0.4 | 0.7 | 0.6 | 0.4 | 0.9 | 0.8 | 0.2 | 0.2 | 0.3 | 0.2 |
| Gender | 0.1 |  | 0.2 | 0.7 | 0.3 | 0.8 | 0.1 | 0.3 | 0.5 | 0.2 | 0.3 | 0.8 | 0.7 | 0.5 | 0.6 | 0.9 | 0.2 | 0.9 | 0.6 | 1 | 0.8 | 0.3 | 0.9 | 0.4 | 0.5 | 0.6 | 0.1 | 0.8 | 0.6 | 0.4 | 0.5 | 1 | 0.2 | 0.2 | 0.5 | 0.6 | 0.4 | 0.5 | 0.7 | 0.1 | 0.6 | 0.6 | 0.2 | 0.7 | 0.2 | 0.9 | 0.3 | 0.9 | 0.7 | 0.1 | 1 | 0.2 |
| BMI | 0.3 | 0.2 |  | 0.4 | 0.3 | 0.9 | 0.3 | 0.7 | 0.4 | 0.7 | 0.6 | 0.5 | 1 | 0.8 | 0.5 | 0.9 | 0.8 | 0.2 | 0.5 | 0.3 | 0.1 | 0.4 | 0.8 | 0.4 | 0.6 | 0.4 | 0.3 | 0.1 | 0.4 | 0.9 | 0.5 | 0.2 | 0 | 0.5 | 0.2 | 0.8 | 0.8 | 0.5 | 0.5 | 0.6 | 0.7 | 0.1 | 0.3 | 0.9 | 0.8 | 0.1 | 0.2 | 0.6 | 0.9 | 0.9 | 0.1 | 0.1 |
| Diabetes | 0.2 | 0.7 | 0.4 |  | 0.3 | 0.3 | 0.3 | 0.6 | 0.6 | 0.3 | 0.3 | 0 | 0.5 | 1 | 0.4 | 0.3 | 0 | 0 | 0.5 | 0.4 | 0 | 0.3 | 0.9 | 0.8 | 0.1 | 0.4 | 0.7 | 0.5 | 0.2 | 0.6 | 0.7 | 0.4 | 0.8 | 0.1 | 0.9 | 0.2 | 0.6 | 0.7 | 0.8 | 0.1 | 0.2 | 0.9 | 1 | 0.9 | 0.1 | 0.6 | 0.6 | 0.6 | 0.7 | 0.3 | 1 | 0.2 |
| Hypertension | 0.1 | 0.3 | 0.3 | 0.3 |  | 0.2 | 0.2 | 0.2 | 0.1 | 0.7 | 0.2 | 0.3 | 0.3 | 0.2 | 0.3 | 0.4 | 0.7 | 0.6 | 0.3 | 0.3 | 0.4 | 0.3 | 0.2 | 0.2 | 0.4 | 0.3 | 0.2 | 0.4 | 0.4 | 0.9 | 0.2 | 0.4 | 0.1 | 0.4 | 0.4 | 0.2 | 0.3 | 0.8 | 0.7 | 0.8 | 0.8 | 0.3 | 0.2 | 0.9 | 0.5 | 0.2 | 0.5 | 0.2 | 0.5 | 0.2 | 0.7 | 0.3 |
| **ob_adult** | **Spearman’s Rho** | | | | | | | | | | | | | | | | | | | | | | | | | | | | | | | | | | | | | | | | | | | | | | | | | | | |
| Age | 1 | -0.1 | -0.4 | 0.3 | 0 | -0.1 | 0.1 | 0.1 | -0.1 | -0.1 | 0.2 | -0.2 | 0.1 | 0.1 | -0.1 | -0.2 | 0 | 0.2 | 0 | 0.1 | 0.2 | 0.1 | 0 | 0.2 | -0.2 | -0.3 | 0.2 | 0 | 0 | 0 | 0 | 0 | 0.3 | 0.2 | -0.1 | 0.2 | -0.1 | 0.2 | 0.3 | 0.3 | 0.4 | -0.4 | -0.3 | 0 | 0.1 | -0.2 | 0.1 | 0.2 | 0.1 | -0.1 | 0.2 | 0.1 |
| Gender | -0.1 | 1 | -0.3 | -0.3 | 0.1 | 0.4 | 0.2 | -0.1 | -0.2 | 0.2 | -0.3 | -0.2 | 0 | 0.3 | -0.1 | -0.2 | -0.1 | -0.4 | 0.2 | 0.1 | 0.2 | -0.1 | -0.1 | **-0.5** | -0.1 | -0.2 | 0 | -0.4 | 0.1 | 0.2 | -0.3 | -0.3 | 0.1 | 0.2 | 0.3 | 0.3 | 0.4 | 0.1 | **-0.5** | -0.4 | -0.1 | -0.2 | -0.1 | 0.1 | -0.2 | -0.3 | 0.1 | -0.1 | 0.1 | 0.2 | 0.3 | 0.2 |
| BMI | -0.4 | -0.3 | 1 | **0.5** | -0.1 | -0.2 | -0.3 | -0.3 | -0.1 | -0.1 | 0 | 0 | -0.4 | **-0.6** | -0.3 | 0 | 0.1 | 0.1 | -0.2 | -0.3 | -0.3 | -0.2 | 0.1 | 0.3 | -0.1 | -0.1 | -0.3 | **0.5** | **-0.6** | 0 | -0.1 | 0 | -0.1 | -0.3 | -0.2 | -0.1 | 0 | **-0.5** | 0.3 | 0 | -0.1 | 0 | -0.1 | -0.1 | 0.4 | 0 | -0.3 | -0.4 | -0.4 | **-0.4** | -0.3 | -0.4 |
| Diabetes | 0.3 | -0.3 | **0.5** | 1 | -0.2 | -0.3 | -0.2 | -0.4 | 0 | -0.4 | **0.5** | 0 | -0.3 | -0.4 | -0.3 | -0.1 | 0 | **0.5** | -0.3 | **-0.6** | -0.3 | 0 | 0.4 | **0.5** | -0.3 | -0.2 | -0.4 | 0.4 | -0.4 | -0.1 | -0.1 | 0.1 | 0.1 | -0.2 | -0.1 | 0.1 | -0.3 | **-0.5** | **0.5** | 0.3 | 0 | -0.2 | -0.2 | 0 | 0.2 | -0.1 | -0.3 | -0.4 | -0.3 | **-0.5** | 0 | -0.3 |
| Hypertension | 0 | 0.1 | -0.1 | -0.2 | 1 | 0.1 | -0.3 | -0.1 | 0 | 0.3 | -0.1 | 0 | -0.3 | -0.2 | 0 | **-0.5** | 0 | -0.4 | **-0.5** | 0.1 | -0.2 | -0.3 | 0.3 | 0 | -0.3 | 0.3 | 0.1 | -0.1 | -0.1 | 0.1 | -0.3 | -0.2 | 0.3 | -0.3 | 0 | -0.3 | 0 | 0 | -0.3 | 0 | -0.3 | -0.1 | 0.2 | -0.2 | 0.1 | 0.3 | -0.3 | -0.1 | -0.2 | 0.4 | **-0.5** | 0 |
| **ob_adult** | **P values** | | | | | | | | | | | | | | | | | | | | | | | | | | | | | | | | | | | | | | | | | | | | | | | | | | | |
| Age |  | 0.8 | 0.1 | 0.3 | 0.9 | 0.8 | 0.6 | 0.8 | 0.8 | 0.6 | 0.3 | 0.5 | 0.7 | 0.8 | 0.8 | 0.5 | 0.9 | 0.3 | 1 | 0.8 | 0.3 | 0.7 | 1 | 0.4 | 0.4 | 0.2 | 0.4 | 1 | 0.9 | 0.9 | 0.9 | 0.9 | 0.2 | 0.3 | 0.7 | 0.3 | 0.7 | 0.3 | 0.2 | 0.2 | 0.1 | 0.1 | 0.3 | 0.9 | 0.6 | 0.5 | 0.8 | 0.5 | 0.8 | 0.8 | 0.3 | 0.6 |
| Gender | 0.8 |  | 0.2 | 0.2 | 0.8 | 0.1 | 0.5 | 0.8 | 0.5 | 0.3 | 0.2 | 0.4 | 0.9 | 0.2 | 0.7 | 0.4 | 0.8 | 0.1 | 0.5 | 0.7 | 0.4 | 0.6 | 0.8 | 0 | 0.8 | 0.5 | 0.9 | 0.1 | 0.6 | 0.3 | 0.2 | 0.2 | 0.7 | 0.5 | 0.2 | 0.2 | 0.1 | 0.8 | 0 | 0.1 | 0.8 | 0.4 | 0.8 | 0.6 | 0.3 | 0.2 | 0.8 | 0.8 | 0.6 | 0.5 | 0.2 | 0.5 |
| BMI | 0.1 | 0.2 |  | 0 | 0.6 | 0.3 | 0.2 | 0.1 | 0.8 | 0.6 | 0.9 | 0.9 | 0.1 | 0 | 0.1 | 1 | 0.6 | 0.8 | 0.4 | 0.2 | 0.2 | 0.3 | 0.6 | 0.2 | 0.6 | 0.6 | 0.2 | 0 | 0 | 0.9 | 0.6 | 0.9 | 0.7 | 0.2 | 0.5 | 0.7 | 0.8 | 0 | 0.2 | 1 | 0.8 | 1 | 0.6 | 0.6 | 0.1 | 0.9 | 0.2 | 0.1 | 0.1 | 0 | 0.2 | 0.1 |
| Diabetes | 0.3 | 0.2 | 0 |  | 0.4 | 0.2 | 0.5 | 0.1 | 0.9 | 0.1 | 0 | 1 | 0.2 | 0.1 | 0.2 | 0.8 | 0.9 | 0 | 0.2 | 0 | 0.2 | 0.9 | 0.1 | 0 | 0.2 | 0.5 | 0.1 | 0.1 | 0.1 | 0.8 | 0.6 | 0.7 | 0.6 | 0.5 | 0.6 | 0.8 | 0.2 | 0 | 0 | 0.1 | 0.9 | 0.5 | 0.3 | 0.9 | 0.4 | 0.6 | 0.2 | 0.1 | 0.2 | 0 | 0.9 | 0.2 |
| Hypertension | 0.9 | 0.8 | 0.6 | 0.4 |  | 0.6 | 0.2 | 0.6 | 0.9 | 0.2 | 0.8 | 1 | 0.3 | 0.5 | 0.9 | 0 | 0.9 | 0.1 | 0 | 0.7 | 0.4 | 0.3 | 0.3 | 1 | 0.3 | 0.2 | 0.6 | 0.7 | 0.5 | 0.6 | 0.2 | 0.4 | 0.2 | 0.2 | 0.9 | 0.3 | 0.9 | 0.9 | 0.1 | 0.9 | 0.2 | 0.7 | 0.4 | 0.3 | 0.8 | 0.2 | 0.2 | 0.6 | 0.4 | 0.1 | 0 | 0.9 |

**Table S10.** Spearman’s correlation table on OTUs and anamnestic and clinical data in CTRL_ado and in CTRL_adult groups.

|  |  |  |  | Actinobacteria | Actinobacteria | Actinobacteria | Actinobacteria | Actinobacteria | Bacteroidetes | Bacteroidetes | Bacteroidetes | Bacteroidetes | Bacteroidetes | Bacteroidetes | Bacteroidetes | Bacteroidetes | Bacteroidetes | Bacteroidetes | Bacteroidetes | Firmicutes | Firmicutes | Firmicutes | Firmicutes | Firmicutes | Firmicutes | Firmicutes | Firmicutes | Firmicutes | Firmicutes | Firmicutes | Firmicutes | Firmicutes | Firmicutes | Firmicutes | Firmicutes | Firmicutes | Firmicutes | Firmicutes | Firmicutes | Firmicutes | Firmicutes | Firmicutes | Firmicutes | Firmicutes | Firmicutes | Firmicutes | Firmicutes | Proteobacteria | Proteobacteria | Verrucomicrobia |
| --- | --- | --- | --- | --- | --- | --- | --- | --- | --- | --- | --- | --- | --- | --- | --- | --- | --- | --- | --- | --- | --- | --- | --- | --- | --- | --- | --- | --- | --- | --- | --- | --- | --- | --- | --- | --- | --- | --- | --- | --- | --- | --- | --- | --- | --- | --- | --- | --- | --- | --- |
|  | Age | Gender | BMI | *Actinomyces* | *Adlercreutzia* | *Collinsella aerofaciens* | Coriobacteriaceae | *Propionibacterium acnes* | Bacteroidaceae | *Bacteroides* | *Bacteroides caccae* | *Bacteroides fragilis* | Barnesiellaceae | *Butyricimonas* | *Parabacteroides* | *Parabacteroides distasonis* | *Paraprevotella* | *Prevotella* | *Prevotella copri* | *Acidaminococcus* | *Anaerostipes* | *Blautia* | Christensenellaceae | Clostridiaceae | *Clostridium* | *Coprococcus* | *Dehalobacterium* | *Dialister* | *Dorea* | *Enterococcus* | Erysipelotrichaceae | *Eubacterium biforme* | *Faecalibacterium prausnitzii* | Gemellaceae | *Granulicatella* | *Lachnospira* | Lachnospiraceae | Mogibacteriaceae | *Oscillospira* | *Phascolarctobacterium* | Rikenellaceae | Ruminococcaceae | *Ruminococcus* | *Ruminococcus gnavus* | *Streptococcus* | *Turicibacter* | *Veillonella parvula* | Enterobacteriaceae | *Sutterella* | *Akkermansia muciniphila* |
| **CTRL_ado** | **Spearman’s Rho** | | | | | | | | | | | | | | | | | | | | | | | | | | | | | | | | | | | | | | | | | | | | | | | | | |
| Age | 1 | 0.1 | **0.8** | 0 | 0 | 0 | 0.2 | 0.1 | -0.2 | -0.2 | 0.2 | -0.1 | -0.2 | -0.1 | 0.2 | -0.1 | -0.3 | 0.3 | 0.1 | -0.1 | -0.4 | -0.5 | -0.1 | -0.2 | 0 | -0.2 | -0.3 | 0.1 | -0.2 | 0 | -0.4 | 0.3 | **-0.6** | -0.2 | -0.2 | -0.1 | -0.2 | 0.1 | -0.2 | -0.3 | -0.2 | 0.5 | -0.2 | -0.1 | -0.3 | -0.3 | 0.4 | -0.1 | -0.3 | -0.2 |
| Gender | 0.1 | 1 | 0 | 0.4 | 0.4 | 0.2 | 0.4 | **0.7** | 0.2 | -0.2 | 0.2 | 0.1 | 0.2 | 0.2 | 0.4 | 0.1 | 0.1 | 0.3 | 0.2 | 0.1 | 0.1 | -0.1 | 0.1 | 0.3 | -0.1 | 0.4 | 0.3 | -0.3 | 0.5 | 0.4 | **0.6** | 0.2 | 0.1 | 0.5 | 0.5 | 0.3 | 0.1 | 0.4 | 0.1 | 0.6 | 0 | 0.5 | -0.1 | 0.4 | 0.1 | 0.2 | 0.6 | **0.7** | 0.1 | 0.6 |
| BMI | **0.8** | 0 | 1 | 0.1 | 0.1 | 0 | 0.3 | 0.1 | -0.3 | -0.1 | 0.2 | 0 | -0.4 | -0.1 | 0.1 | -0.2 | -0.2 | 0.2 | 0.4 | 0 | -0.3 | -0.4 | -0.1 | 0 | 0 | 0 | -0.1 | 0.3 | -0.3 | 0.1 | -0.5 | 0.3 | **-0.8** | -0.3 | -0.3 | 0 | -0.2 | 0.1 | -0.1 | -0.5 | -0.3 | 0.2 | 0 | -0.1 | 0 | -0.4 | 0.6 | -0.2 | -0.3 | -0.1 |
| **CTRL_ado** | **P values** | | | | | | | | | | | | | | | | | | | | | | | | | | | | | | | | | | | | | | | | | | | | | | | | | |
| Age |  | 0.8 | 0 | 0.9 | 0.9 | 1 | 0.6 | 0.7 | 0.4 | 0.6 | 0.6 | 0.7 | 0.4 | 0.7 | 0.6 | 0.8 | 0.3 | 0.3 | 0.7 | 0.7 | 0.2 | 0.1 | 0.8 | 0.5 | 0.9 | 0.5 | 0.4 | 0.8 | 0.6 | 0.9 | 0.2 | 0.4 | 0 | 0.6 | 0.6 | 0.7 | 0.5 | 0.7 | 0.5 | 0.3 | 0.6 | 0.1 | 0.5 | 0.7 | 0.3 | 0.3 | 0.3 | 0.7 | 0.4 | 0.6 |
| Gender | 0.8 |  | 1 | 0.3 | 0.3 | 0.5 | 0.2 | 0 | 0.6 | 0.5 | 0.5 | 0.8 | 0.5 | 0.6 | 0.2 | 0.9 | 0.8 | 0.4 | 0.5 | 0.8 | 0.9 | 0.9 | 0.9 | 0.3 | 0.8 | 0.3 | 0.4 | 0.4 | 0.1 | 0.3 | 0 | 0.5 | 0.8 | 0.1 | 0.1 | 0.3 | 0.9 | 0.2 | 0.8 | 0.1 | 1 | 0.1 | 0.8 | 0.2 | 0.9 | 0.5 | 0.1 | 0 | 0.8 | 0.1 |
| BMI | 0 | 1 |  | 0.7 | 0.8 | 0.9 | 0.4 | 0.7 | 0.3 | 0.7 | 0.5 | 0.9 | 0.2 | 0.6 | 0.7 | 0.6 | 0.5 | 0.4 | 0.2 | 0.9 | 0.3 | 0.2 | 0.8 | 0.9 | 1 | 1 | 0.7 | 0.4 | 0.3 | 0.8 | 0.1 | 0.4 | 0 | 0.4 | 0.4 | 0.9 | 0.6 | 0.8 | 0.6 | 0.1 | 0.4 | 0.5 | 0.9 | 0.8 | 0.9 | 0.2 | 0.1 | 0.6 | 0.4 | 0.7 |
| **CTRL_adult** | **Spearman’s Rho** | | | | | | | | | | | | | | | | | | | | | | | | | | | | | | | | | | | | | | | | | | | | | | | | | |
| Age | 1 | 0.2 | 0.2 | 0.2 | 0.1 | 0.3 | 0.1 | 0.3 | -0.2 | 0 | 0.1 | 0.4 | -0.2 | -0.1 | -0.1 | -0.1 | 0.3 | 0.2 | 0.5 | 0.2 | -0.4 | -0.1 | 0.3 | -0.2 | -0.1 | -0.1 | 0.2 | -0.2 | 0.5 | 0.2 | -0.1 | 0.5 | 0.1 | 0.2 | 0.3 | 0.3 | 0.4 | 0.1 | 0.2 | -0.1 | -0.4 | -0.1 | 0.2 | 0.3 | 0.2 | 0.3 | 0.1 | -0.4 | -0.2 | -0.1 |
| Gender | 0.2 | 1 | 0.3 | 0.4 | 0.2 | 0.4 | -0.2 | 0.4 | -0.4 | 0.2 | 0.4 | 0.2 | 0.3 | 0.5 | -0.1 | 0.5 | 0.5 | 0.3 | 0.5 | 0.4 | 0.1 | -0.6 | 0.4 | 0.3 | 0.6 | -0.4 | 0.5 | 0.4 | -0.6 | 0.4 | 0.3 | 0.1 | 0.1 | 0.4 | 0 | 0.4 | -0.2 | -0.3 | -0.1 | 0.2 | 0.2 | 0.4 | 0.2 | 0.1 | -0.2 | 0.1 | 0.2 | 0.4 | 0.6 | -0.1 |
| BMI | 0.2 | 0.3 | 1 | 0.3 | 0.2 | 0.1 | -0.5 | 0.1 | -0.6 | 0.2 | 0.2 | 0.1 | 0 | 0 | -0.5 | 0.2 | 0.4 | 0.5 | 0.6 | 0.3 | 0.2 | 0.1 | 0.2 | 0.2 | 0.4 | -0.2 | -0.1 | 0 | -0.2 | 0.3 | 0 | 0.1 | 0 | 0.3 | -0.4 | 0.1 | 0 | -0.3 | 0 | 0.2 | -0.1 | -0.1 | -0.2 | 0.3 | -0.4 | 0.3 | 0.2 | 0.3 | 0.3 | -0.8 |
| **CTRL_adult** | **P values** | | | | | | | | | | | | | | | | | | | | | | | | | | | | | | | | | | | | | | | | | | | | | | | | | |
| Age |  | 0.5 | 0.5 | 0.5 | 0.7 | 0.4 | 0.8 | 0.3 | 0.6 | 1 | 0.8 | 0.3 | 0.5 | 0.7 | 0.7 | 0.8 | 0.3 | 0.5 | 0.1 | 0.5 | 0.2 | 0.9 | 0.3 | 0.5 | 0.8 | 0.8 | 0.6 | 0.5 | 0.1 | 0.5 | 0.7 | 0.1 | 0.9 | 0.5 | 0.3 | 0.3 | 0.2 | 0.8 | 0.6 | 0.8 | 0.2 | 0.8 | 0.6 | 0.4 | 0.6 | 0.4 | 0.7 | 0.2 | 0.6 | 0.7 |
| Gender | 0.5 |  | 0.3 | 0.3 | 0.5 | 0.2 | 0.5 | 0.2 | 0.2 | 0.6 | 0.2 | 0.5 | 0.3 | 0.1 | 0.8 | 0.1 | 0.1 | 0.3 | 0.1 | 0.3 | 0.8 | 0 | 0.3 | 0.4 | 0 | 0.2 | 0.1 | 0.2 | 0.1 | 0.3 | 0.4 | 0.9 | 0.9 | 0.3 | 1 | 0.2 | 0.6 | 0.3 | 0.9 | 0.5 | 0.6 | 0.2 | 0.6 | 0.8 | 0.5 | 0.8 | 0.5 | 0.3 | 0 | 0.9 |
| BMI | 0.5 | 0.3 |  | 0.4 | 0.6 | 0.7 | 0.1 | 0.6 | 0.1 | 0.5 | 0.5 | 0.8 | 0.9 | 0.9 | 0.1 | 0.5 | 0.2 | 0.1 | 0.1 | 0.4 | 0.5 | 0.8 | 0.5 | 0.5 | 0.2 | 0.6 | 0.8 | 0.9 | 0.5 | 0.4 | 0.9 | 0.8 | 1 | 0.4 | 0.3 | 0.6 | 0.9 | 0.4 | 0.9 | 0.4 | 0.7 | 0.6 | 0.6 | 0.3 | 0.2 | 0.3 | 0.6 | 0.3 | 0.3 | 0 |


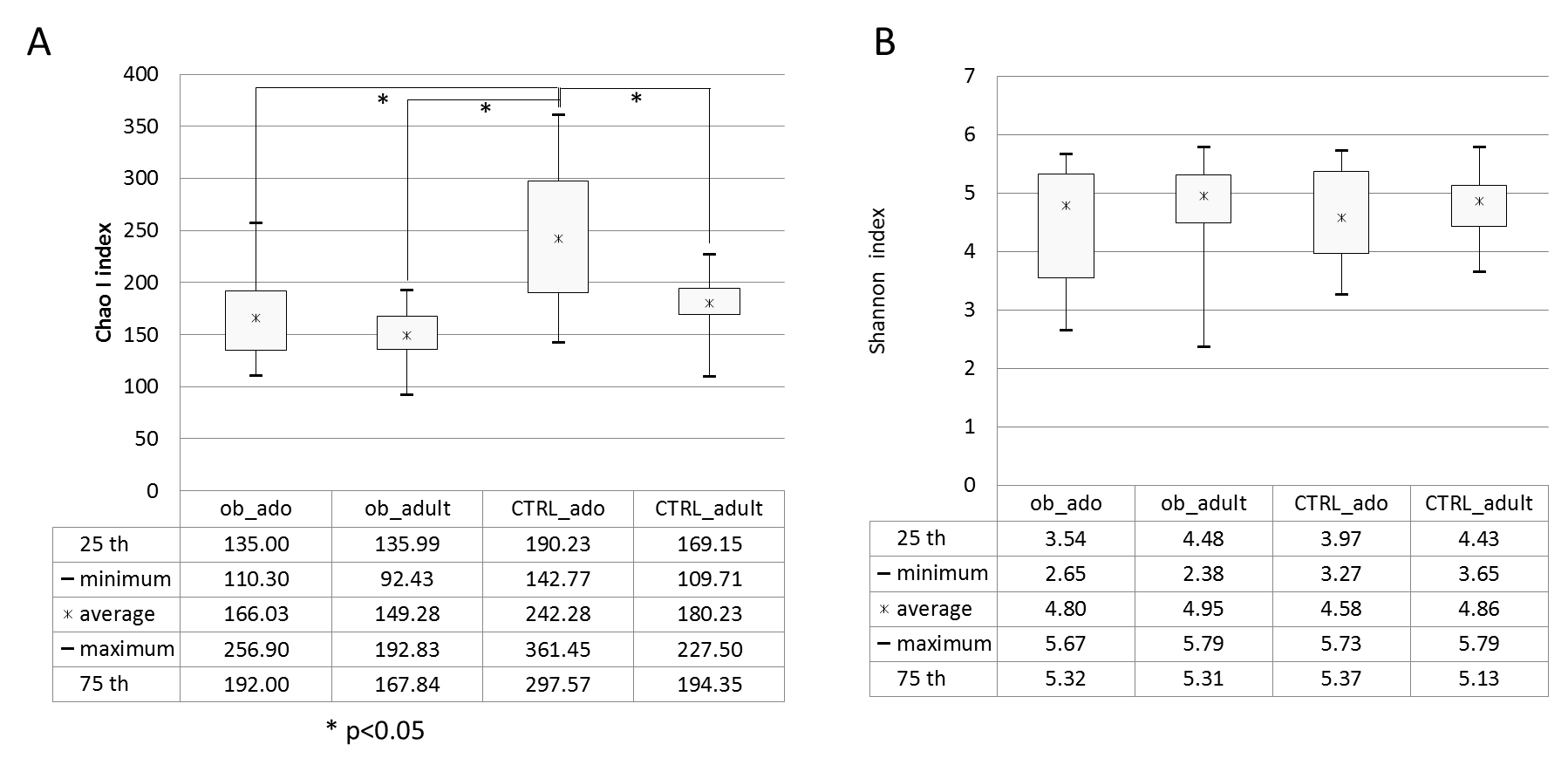


**Figure S1.** **Figure S1.** **Box plots of the mean values of the ChaoI (panel A) and Shannon (panel B) indexes for each patient/CTRL group.** The plot represents the average, minimum, maximum values, 25^th^ and 75^th^ percentiles calculated for CTRL_ado (control adolescent), CTRL_adult (control adult), ob_ado (obese adult), and ob_adult (obese adult) groups. The summary table shows detailed values.

**Figure S2. Bar chart of the selected 12 OTUs in each groups, after Lambda Wilks filtering.** Each bar in the plot represents values of relative abundance of the 12 selected OTUs in CTRL_ado, CTRL_adult, ob_ado and ob_adult groups.

**
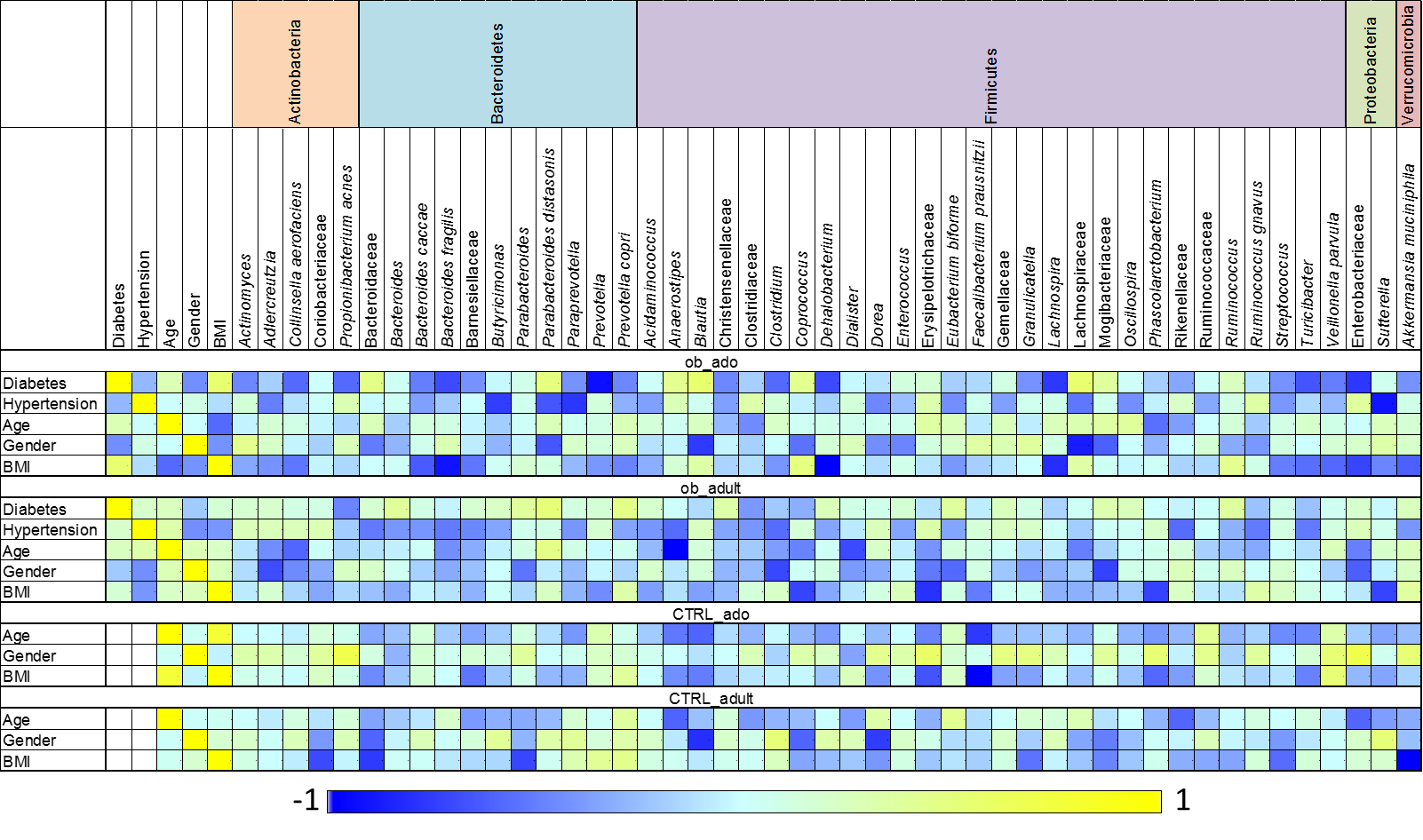
**

**Figure S3. Spearman’s correlation heat-map of OTUs and anamnestic and clinical features.** The correlation levels (represented by colored squares from yellow, 1 to blue, -1) are represented among OTUs. The analysis was performed by Spearman’s test.

**
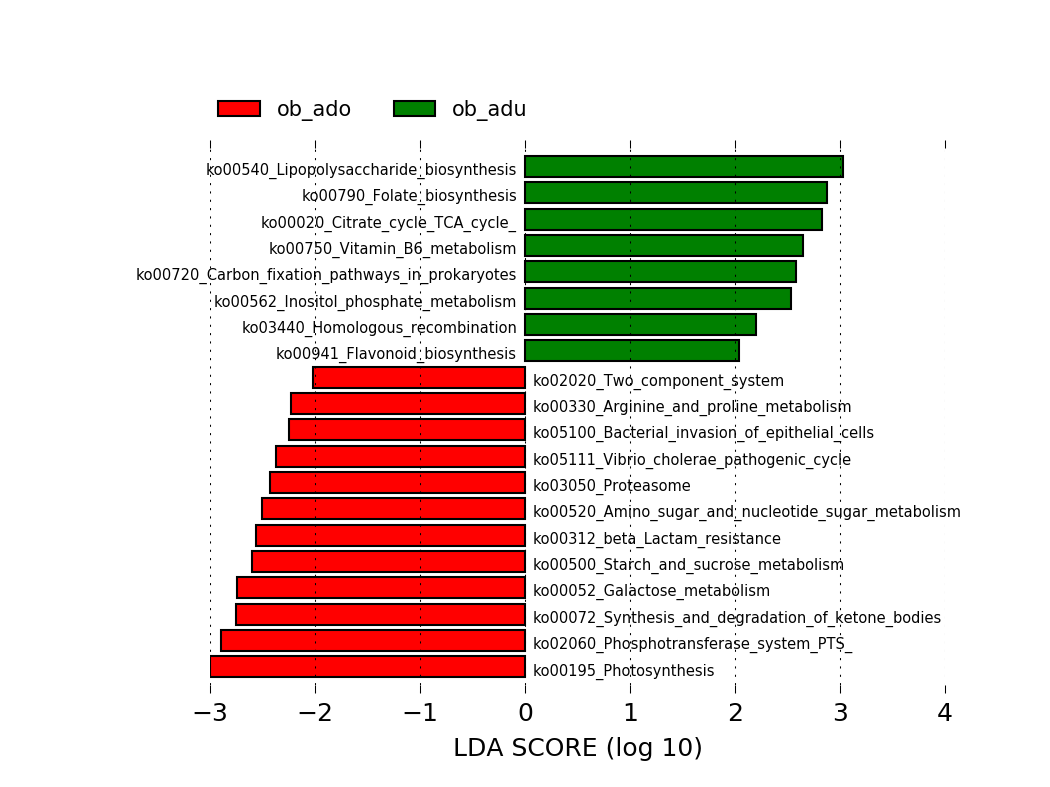
**

**Figure S4.** KEGGs biomarkers associated with adolescent and adult obese. A linear discriminant effect size (LeFse) analysis have been performed (α value = 0.05, logarithmic LDA score threshold = 2.0).


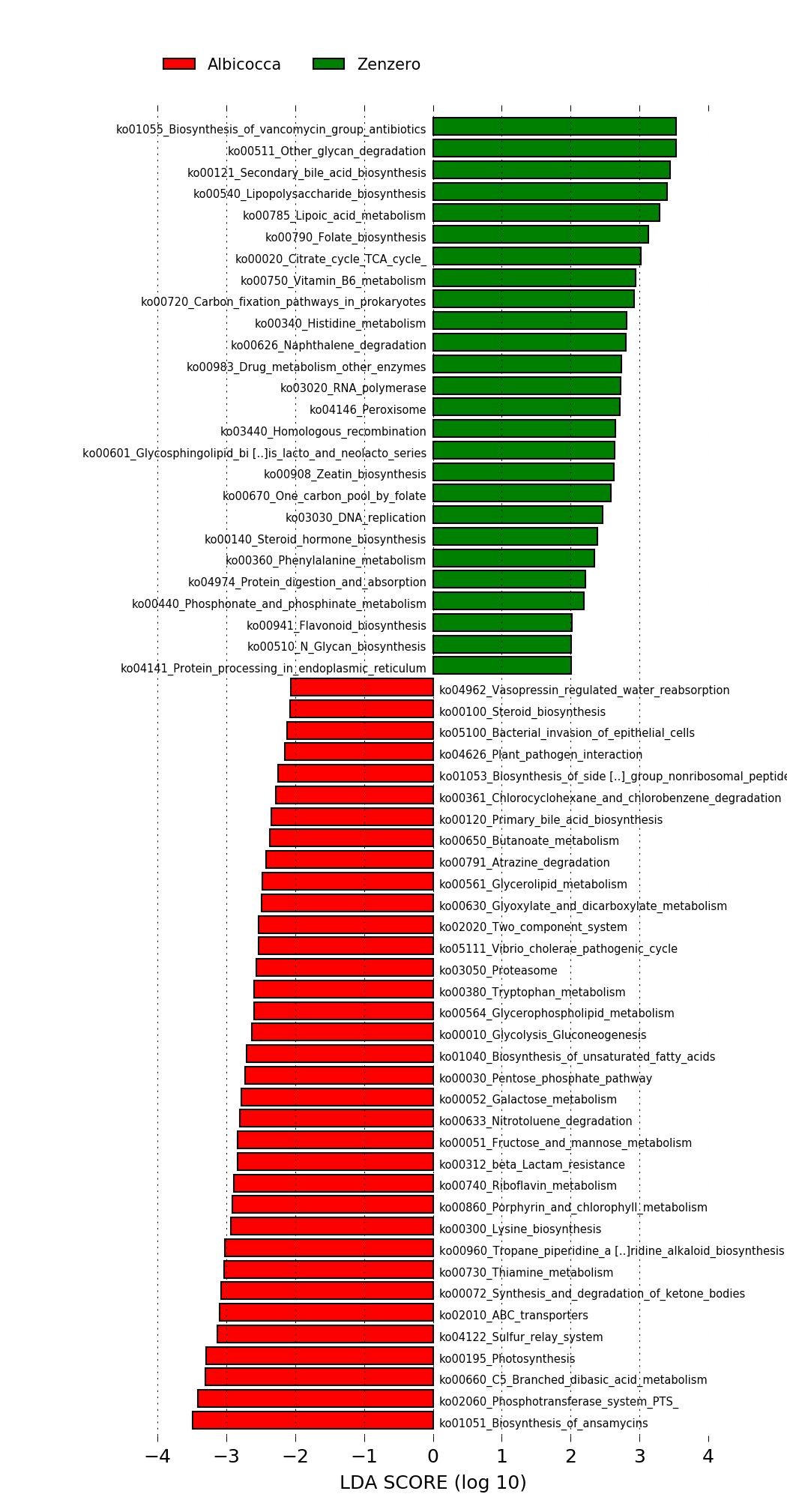

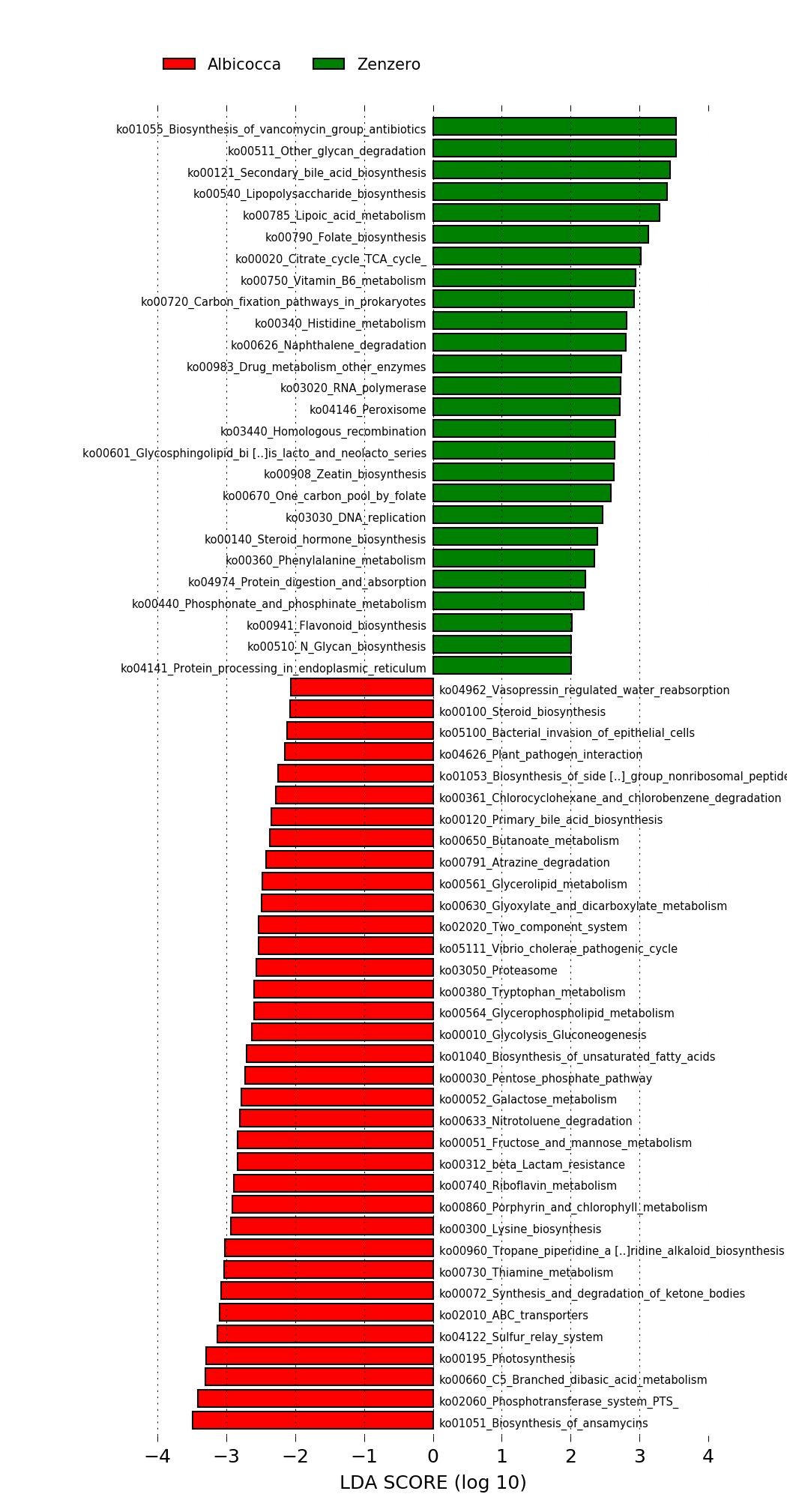

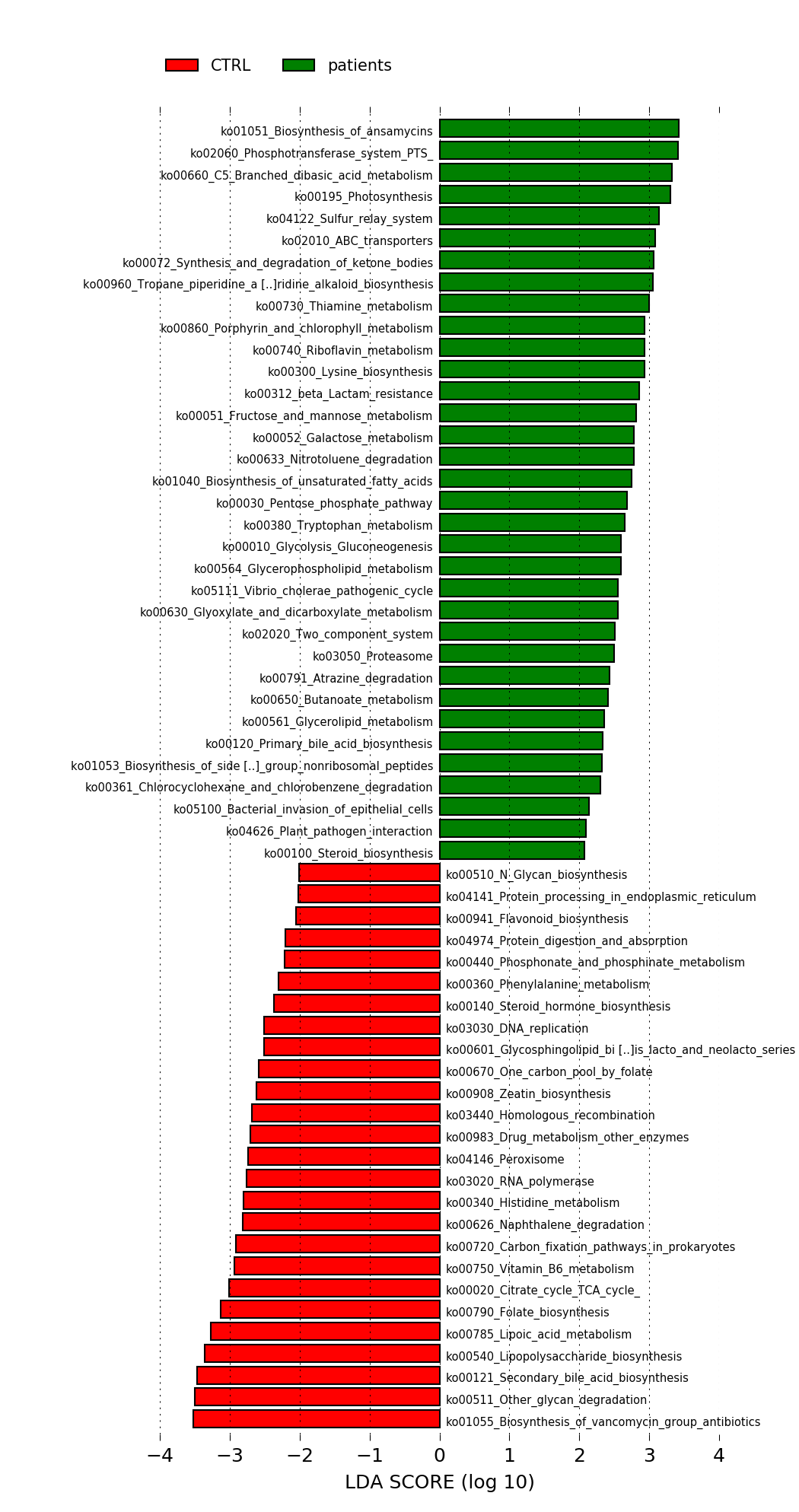

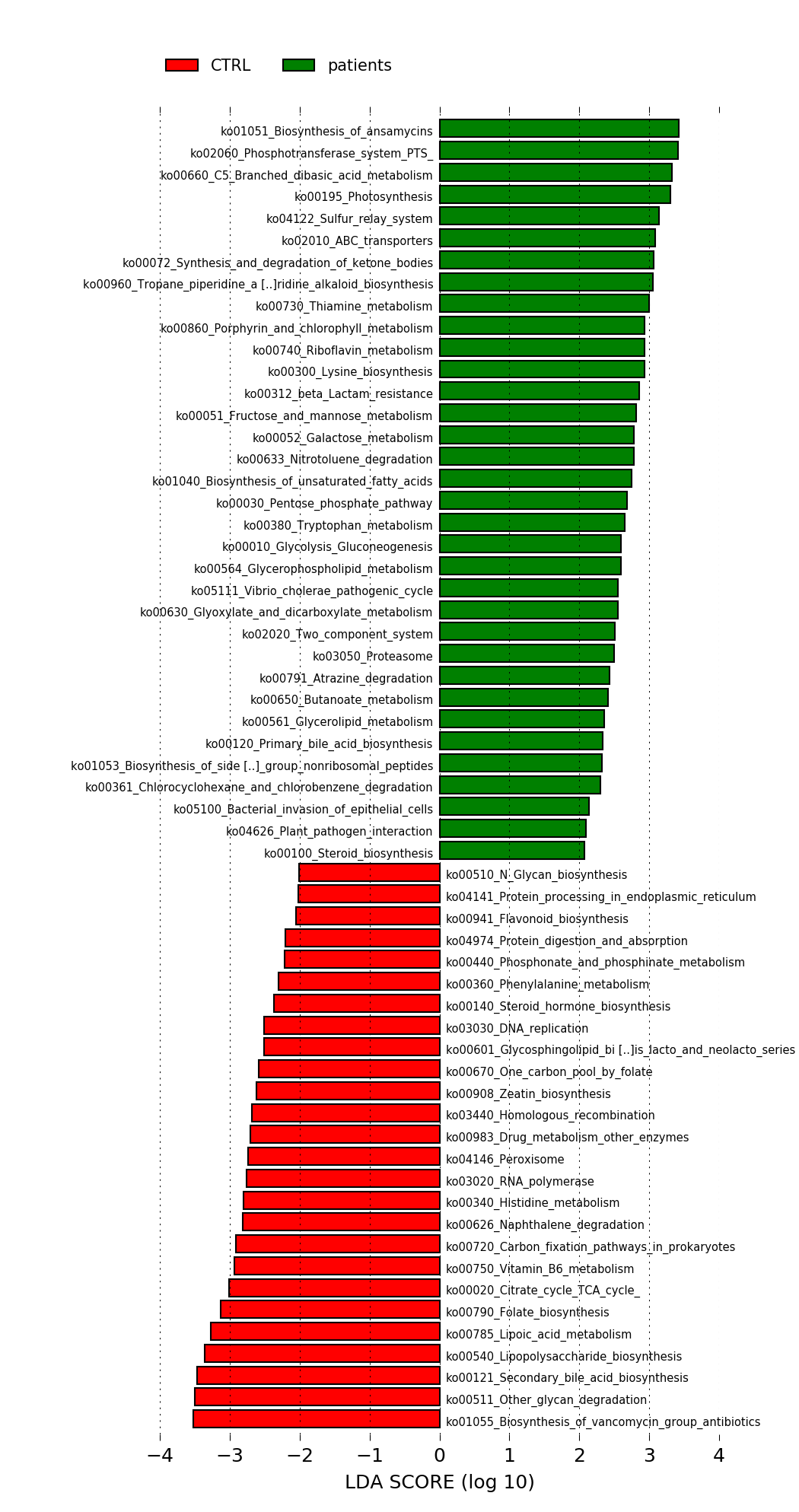

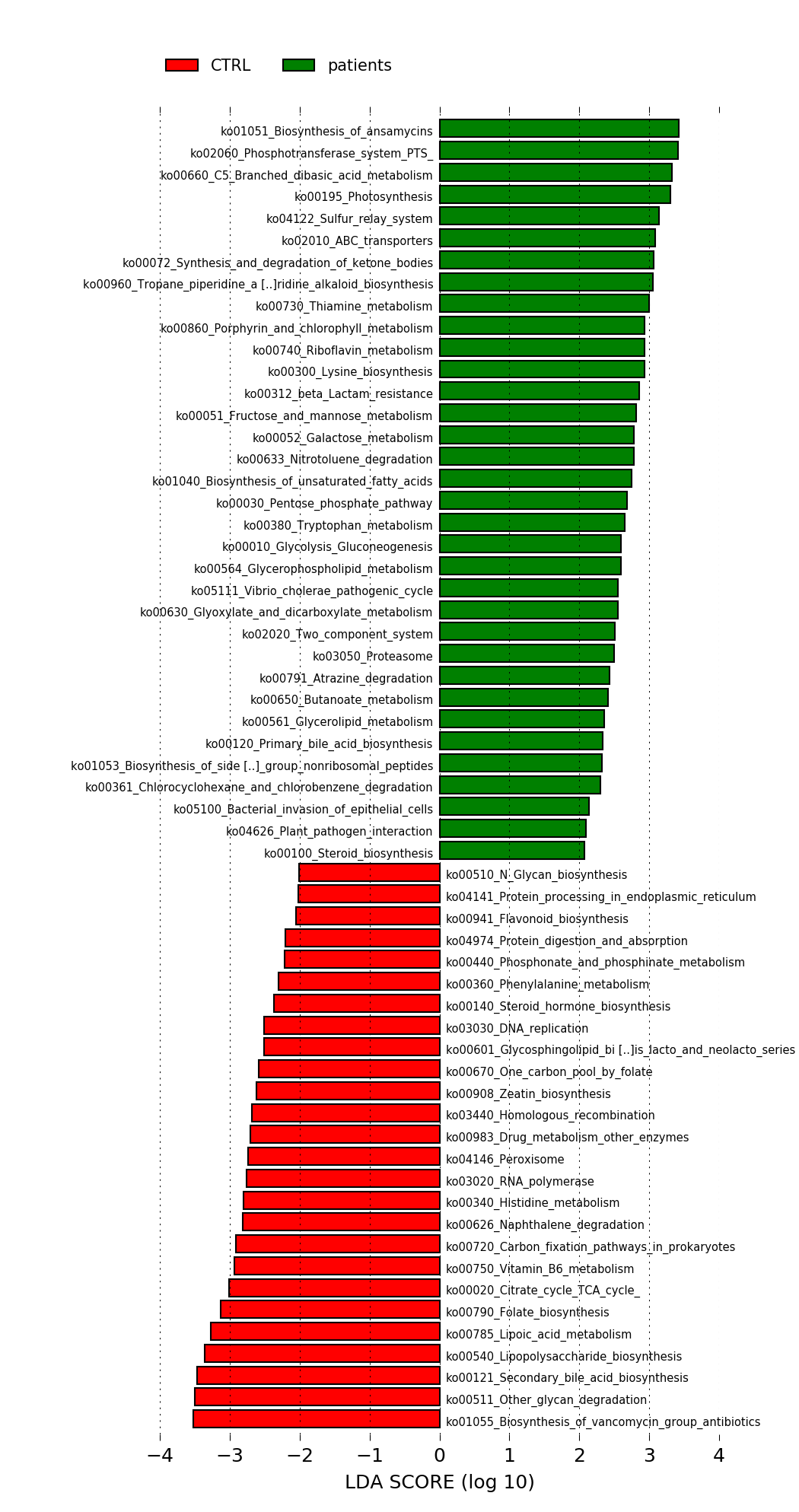

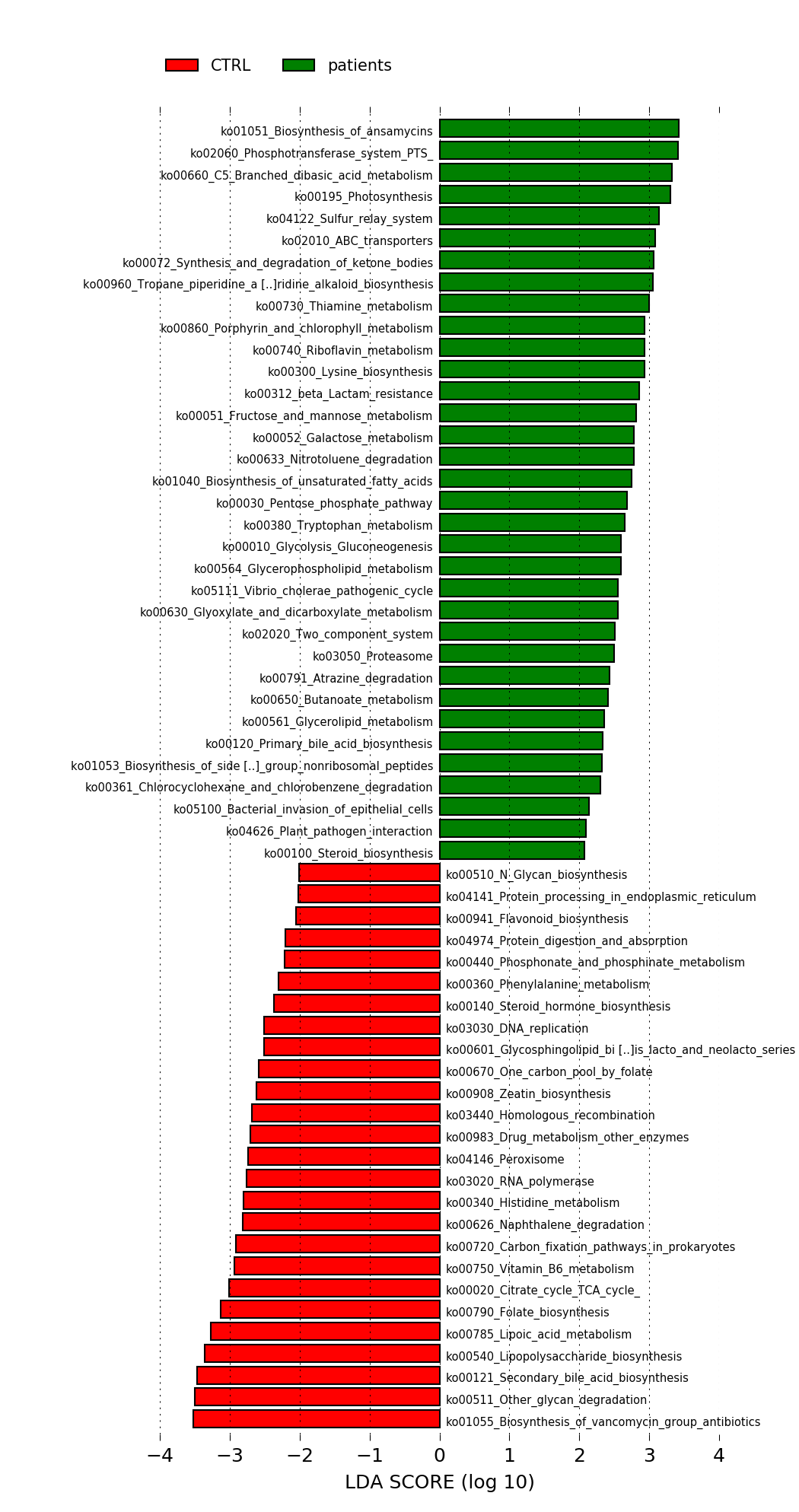

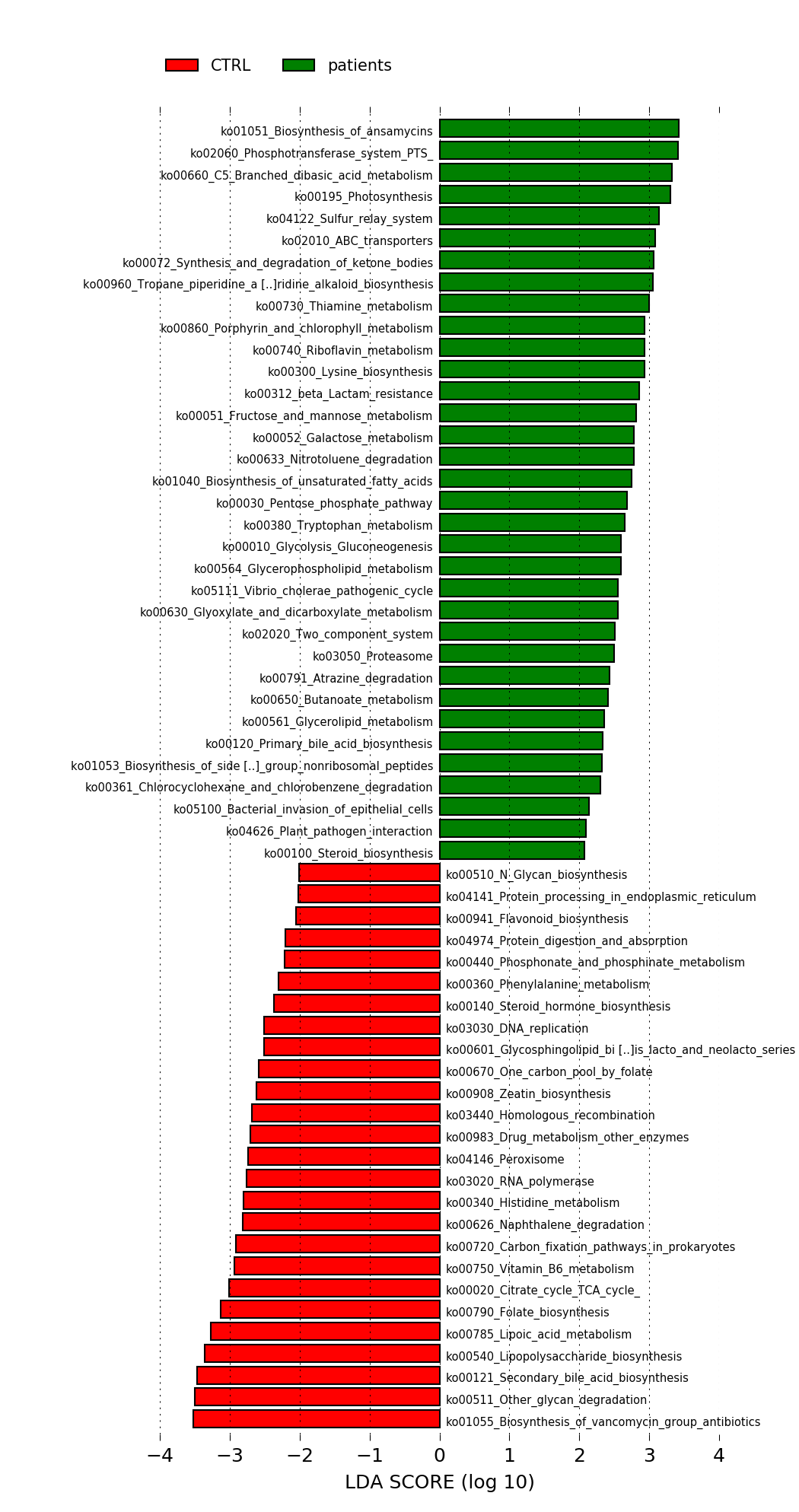


ob_ado


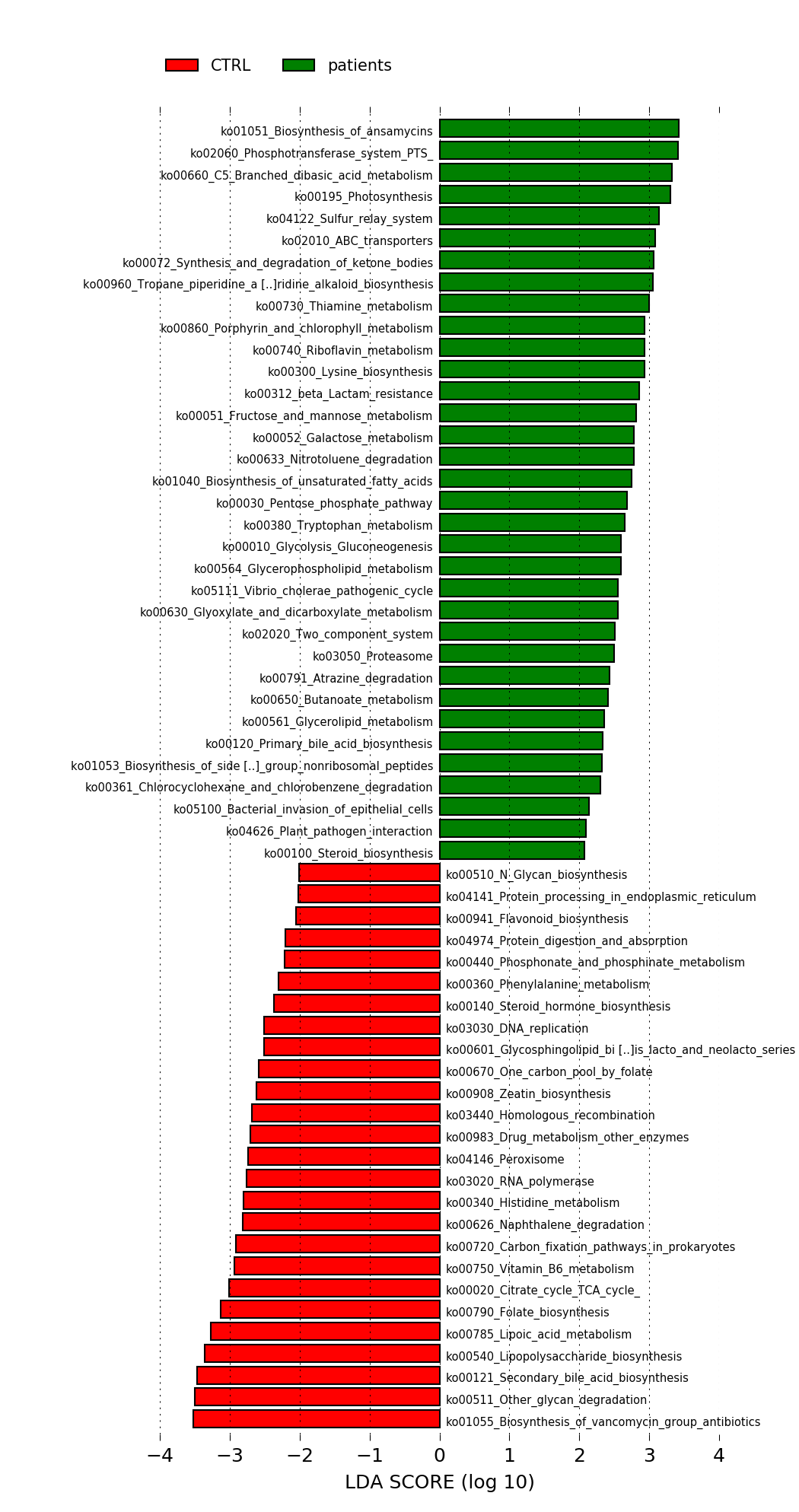


CTRL_ado

**Figure S5.** KEGGs biomarkers associated with adolescent obese and CTRL conditions. A linear discriminant effect size (LeFse) analysis have been performed (α value = 0.05, logarithmic LDA score threshold = 2.0).

**
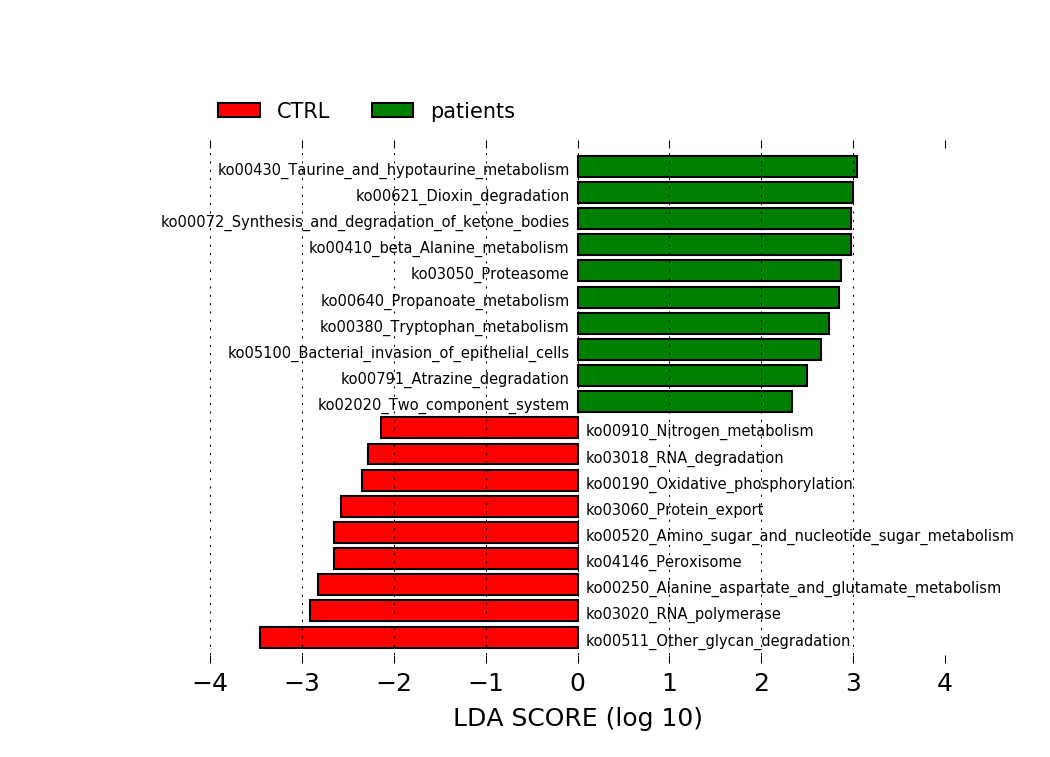

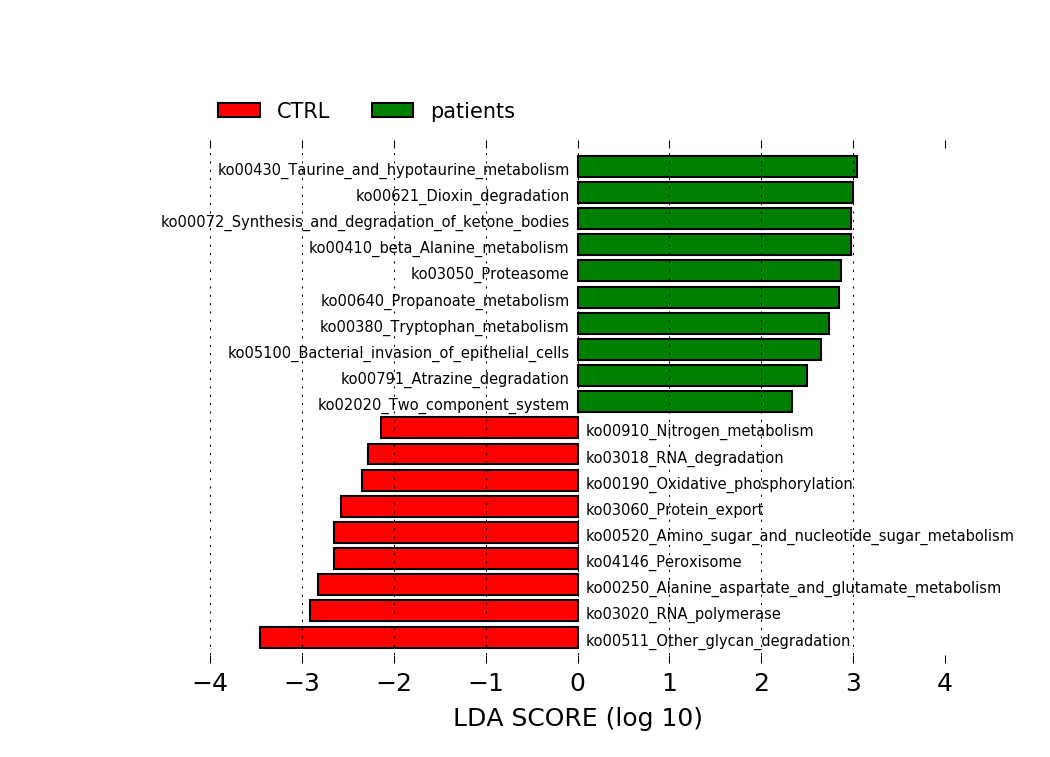
**

CTRL_adult

ob_adult

**
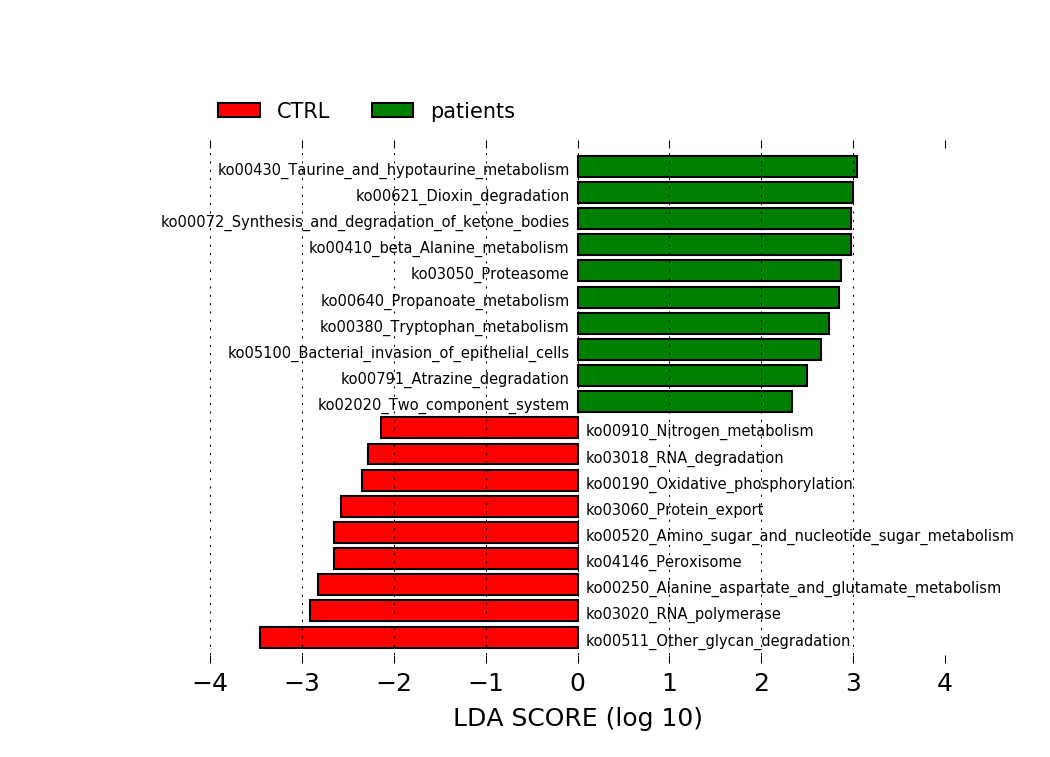
**

**Figure S6.** KEGGs biomarkers associated with adult obese and CTRL conditions. A linear discriminant effect size (LeFse) analysis have been performed (α value = 0.05, logarithmic LDA score threshold = 2.0).
